# Synthesis, characterization and biological activity of methotrexate-derived salts in lung cancer cells†

**DOI:** 10.1039/d4md00960f

**Published:** 2025-04-16

**Authors:** Dário Silva, Sandra Cordeiro, Pedro V. Baptista, Alexandra R. Fernandes, Luis C. Branco

**Affiliations:** a LAQV-REQUIMTE, Nova School of Science and Technology, NOVA University Lisbon 2829-516 Caparica Portugal l.branco@fct.unl.pt; b Associate Laboratory i4HB – Institute for Health and Bioeconomy, NOVA School of Science and Technology, NOVA University Lisbon 2829-516 Caparica Portugal ma.fernandes@fct.unl.pt; c UCIBIO – Applied Molecular Biosciences Unit, Department of Life Sciences, NOVA School of Science and Technology, NOVA University Lisbon 2829-516 Caparica Portugal

## Abstract

Lung cancer is one of the deadliest types of cancer, and is a public health problem worldwide. Methotrexate (MTX), a class IV drug in the biopharmaceutical classification system, is a folate antagonist that has demonstrated efficacy in cancer treatment. A suitable combination of MTX as a di-anion and biocompatible counter ions allowed the modulation of their physicochemical properties. In this work, twelve MTX salts were prepared and characterized by ^1^H NMR, ^13^C NMR, and elemental analysis. The antiproliferative effects of MTX salts were studied in A459 and H1975 (lung cancer cell lines) with three promising results: [C_12_mim]_2_[MTX] (IC_50_ = 0.55 ± 0.25) > [C_10_-3-picoline]_2_[MTX] (IC_50_ = 0.94 ± 0.03) > [C_10_mim]_2_[MTX] (IC_50_ = 1.71 ± 0.23) in A549. These three MTX salts also demonstrated intrinsic apoptosis, avoiding necrosis and the formation of reactive oxygen species.

## Abstract 2

High antiproliferative activity against lung cancer cell lines.

## Graphical abstract

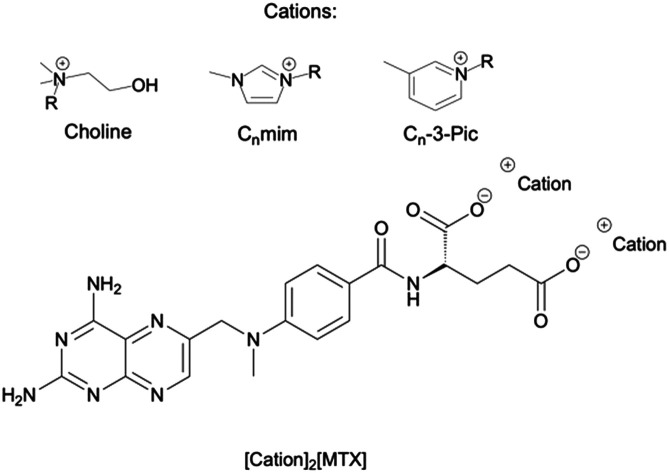

## 1. Introduction

Cancer remains one of the main causes of death worldwide. In 2020, 9.9 million people died around the world and 18% of the deaths were due to lung cancer, which is one of the deadliest types of cancer.^1^ Lung cancer can be divided into two main histological types: small cell lung cancer (SCLC) and non-small cell lung cancer (NSCLC).^2,3^ NSCLC is the most prevalent form of lung cancer with 80–85% incidence. Platinum-based chemotherapy remains the foremost treatment for NSCLC but, due to its toxicity, new forms of treatment, such as targeted therapy or immune checkpoint inhibitors,^3^ have also been considered.

Methotrexate (MTX) is a folate antagonist, which inhibits dihydrofolate reductase, which is essential for cellular replication. It is used in the treatment of different diseases, such as cancer, including NSCLC,^4,5^ rheumatoid arthritis,^6^ and psoriasis.^7^

As a folate antagonist, MTX has already shown efficacy in cancer treatment, especially in cancers with an abundance of folate receptors. Different reported studies showed a higher expression of folate receptors in lung cancer,^8^ which makes MTX a specific drug for lung cancer treatment. However, treatments with high doses of MTX cause significant side effects.^9^ Conversely, MTX also could induce resistance in cancer cells through folate cancer gene downregulation,^10^ leading to a reduction in cellular uptake of MTX as well as its treatment prospects. Additionally, MTX is a class IV drug in the biopharmaceutical classification system, showing low solubility and low permeability and, consequently, poor bioavailability.^11^ Recently, different approaches have been studied to target the delivery of MTX, involving nanocarriers or task-specific peptides, which selectively penetrate the cells linked to MTX.^12,13^

Ionic liquids (ILs) are low-melting organic salts with tunable physical–chemical properties and have found a large number of applications over the past two decades. Generally, it is possible to consider the evolution of ILs in three generations, with the third focusing on biological and pharmaceutical applications. In the last decade, different groups have reported the possibility of combining active pharmaceutical ingredients (APIs) with biocompatible counter ions forming API-ILs (also classified as active pharmaceutical organic salt & ionic liquids or API-OSILs and a group of uniform materials based on organic salts or GUMBOS). According to the cation–anion combinations, it is possible to tune their properties, such as solubility, permeability, and cytotoxicity^14^ as well as increasing their therapeutic activity. In fact, studies with MTX have already shown an enhancement in solubility and anti-proliferative activity in cancer cell lines when combined with ionic liquids (MTX-ILs).^15,16^ MTX has two ionizable carboxylic acids, meaning that it is a suitable pharmaceutical drug to combine with appropriate counter ions. Taking advantage of our previous experience in designing new API-OSILs with improved physical–chemical and pharmaceutical properties of the APIs, such as ciprofloxacin,^17^ mefloquine,^18^ levothyroxine,^19^ amphotericin B,^20^ valproate,^21^ hydroxyquinoline,^22^ penicillin,^23^ and amoxicilin,^23^ several biocompatible counter ions can be designed to develop new series of MTX salts. Additionally, the biocompatibility and low toxicity of some counter ions, combined with their bioavailability, can improve the original antitumoral activity of MTX while reducing side effects.

**Fig. 1:**
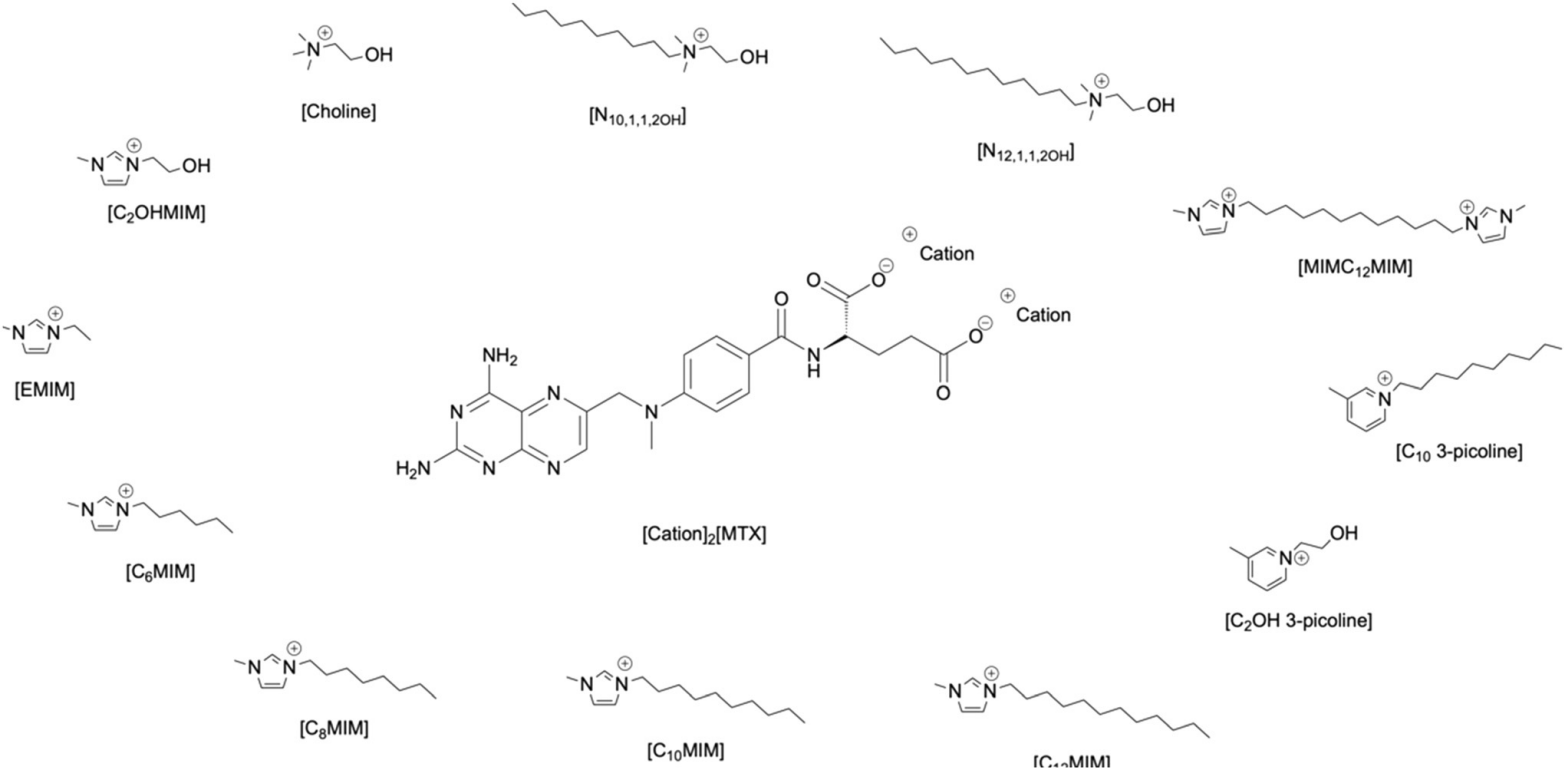
All selected counter-ions combined with methotrexate (MTX) di-anion.

In this context, the main goal of the present study is focused on the development of MTX salts (Fig. 1) and the evaluation of their properties, including bioavailability and cytotoxicity studies, against lung cancer cell lines.

## 2. Experimental

### 2.1 Materials

Methotrexate hydrate (purity >98%) was purchased from TCI. Solvents were purchased from LabChem. The ion-exchange resin Amberlyst A26 (OH) (0.8 meq mL^−1^ ion-exchange capacity) was purchased from Alfa Aesar.

### 2.2 MTX salts and counter ions: synthesis and characterization

#### 2.2.1 MTX salts synthesis

##### General synthesis

Methotrexate (1.0 molar equiv.) was slightly dissolved in 5 mL of distilled water; then, a counter ion (2.0 molar equiv.), previously dissolved in 5 mL of water and treated with Amberlyst A-26 (1 h), was added and the reaction mixture was stirred at 40 °C for 2 h. The solvent was removed under reduced pressure to obtain the desired product.

###### [Na]_2_[MTX]

Following the general procedure, NaOH (17.6 mg; 0.44 mmol) was added to a methotrexate solution. Yellow solid (96% w/w).

^1^H NMR (400.13 MHz, D_2_O) *δ* (ppm): 8.45 (s, 1H); 7.63 (d, *J* = 8.90 Hz, 2H); 6.73 (d, *J* = 9.10 Hz, 2H); 4.58 (s, 2H); 4.29 (dd, *J*_1_ = 8.5 Hz, *J*_2_ = 4.70 Hz, 1H); 3.07 (s, 3H); 2.36–1.94 (m, 4H).

^13^C NMR (100.63 MHz, D_2_O) *δ* (ppm): 182.36; 179.19; 169.28; 162.85; 161.99; 153.36; 151.66; 149.10; 148.08; 128.78; 122.21; 120.58; 111.72; 55.83; 54.83; 38.62; 34.27; 28.57.

###### [Choline]_2_[MTX]

Following the general procedure, [choline][OH] (61.5 mg; 0.44 mmol) was added to a methotrexate solution. The final product was obtained as a yellow wax (128 mg; 88%).

^1^H NMR (400.13 MHz, D_2_O) *δ* (ppm): 8.54 (s, 1H); 7.68 (d, *J* = 7.20 Hz, 2H); 6.83 (d, *J* = 7.20 Hz, 2H); 4.72 (s, 1H); 4.29 (q, *J*_1_ = 7.20 Hz, *J*_2_ = 3.60 Hz, 1H); 4.05–4,02 (m, 4H); 3.50 (t, *J* = 4.40 Hz, 4H); 3.18 (s, 18H); 3.14 (s, 3H); 2.31–2.24 (m, 2H); 2.17–2.10 (m, 1H); 2.05–1.97 (m, 1H).

^13^C NMR (100.63 MHz, D_2_O) *δ* (ppm): 182.24; 179.08; 169.13; 162.83; 161.90; 153.11; 151.61; 149.11; 148.2; 128.78; 122.16; 120.52; 111.69; 67.38; 67.36; 67.33; 55.81; 55.54; 54.81; 53.83; 53.80; 53.77; 38.69; 34.27; 28.57.

IR (ATR) *ν*_max_ (cm^−1^): 3328, 3218, 1589, 1556, 1449, 1380, 1208, 1087, 953, 826.

Elemental analysis calcd for C_30_H_48_N_10_O_7_·8.5H_2_O (%): C 44.27; H 8.05; N 17.21; found: C 44.58; H 7.89; N 17.08.

###### [N_12,1,1,2OH_]_2_ [MTX]

[N_12,1,1,2OH_][Br] was synthesized according to the literature.^24^

Following the general procedure, [N_12,1,1,2OH_][OH] (129 mg; 0.44 mmol) was added to a methotrexate solution. The final product was obtained as a yellow wax (175 mg; 95%).

^1^H NMR (400.13 MHz, D_2_O) *δ* (ppm): 8.50 (s, 1H); 7.74 (d, *J* = 8.3 Hz, 2H); 6.77 (d, *J* = 8.50 Hz, 2H); 4.33 (s, 1H); 3.97 (s, 4H); 3.40 (s, 4H); 3.24–3.13 (m, 6H); 3.05 (s, 12H); 2.31–1.96 (m, 3H); 1.55 (s, 4H) 1.33–1.01 (m, 40H); 0.89 (t, *J* = 7.0 Hz, 6H).

^13^C NMR (100.63 MHz, D_2_O) *δ* (ppm): 181.52; 178.26; 167.11; 162.79; 162.30; 154.14; 150.82; 146.98; 128.85; 122.06; 121.63; 111.51; 64.92; 55.52; 55.29; 51.35; 39.05; 34.24; 32.02; 29.90; 29.84; 29.71; 29.61; 29.55; 29.08; 26.06; 22.69; 22.25; 13.94.

IR (ATR) *ν*_max_ (cm^−1^): 3302, 2921, 2853, 1628, 1556, 1449, 1374, 1208, 1087.

Elemental analysis calcd for C_52_H_92_N_10_O_7_·8H_2_O (%): C 56.09; H 9.78; N 12.58; found: C 55.92; H 10.49; N 12.57.

###### [N_10,1,1,2OH_]_2_[MTX]

[N_10,1,1,2OH_][Br] was synthesized according to the literature.^24^

Following the general procedure, [N_10,1,1,2OH_][OH] (136.6 mg; 0.44 mmol) was added to a methotrexate solution. The final product was obtained as a yellow wax (189 mg; 94%).

^1^H NMR (400.13 MHz, D_2_O) *δ* (ppm): 8.63 (s, 1H); 7.72 (d, *J* = 8.6 Hz, 2H); 6.90 (d, *J* = 8.70 Hz, 2H); 4.31 (dd, *J*_1_ = 8.6 Hz, *J*_2_ = 4.5 Hz, 1H); 4.03 (s, 4H); 3.46 (t, *J* = 6.2 Hz, 4H); 3.36–3.27 (m, 4H); 3.21 (s, 3H); 3.11 (s, 12H); 2.34–1.95 (m, 4H); 1.71 (s, 4H) 1.34–1.11 (m, 30H); 0.84 (t, *J* = 6.8 Hz, 6H).

^13^C NMR (100.63 MHz, D_2_O) *δ* (ppm): 181.88; 178.68; 168.26; 162.90; 162.05; 153.56; 151.34; 149.16; 147.87; 128.83; 122.19; 120.92; 111.71; 65.25; 64.90; 55.62; 55.29; 54.96; 51.29; 38.90; 34.22; 31.41; 28.89; 28.80; 28.77; 28.41; 25.61; 22.24; 21.96; 13.62.

IR (ATR) *ν*_max_ (cm^−1^): 3309, 3182, 2924, 2853, 1628, 1585, 1553, 1445, 1377, 1208, 1090, 830, 761.

Elemental analysis calcd for C_48_H_84_N_10_O_7_·6H_2_O (%): C 56.45; H 9.47; N 13.71; found: C 56.60; H 9.77; N 14.13.

###### [MIMC_12_MIM][MTX]

[MIMC_12_MIM][Br] was synthesized according to the literature.^25^

Following the general procedure, [MIMC_12_MIM][OH] (102.2 mg; 0.22 mmol) was added to a methotrexate solution. The final product was obtained as a yellow wax (168 mg; 97%).

^1^H NMR (400.13 MHz, D_2_O) *δ* (ppm): 8.65 (s, 1H); 8.52 (s, 1H); 7.60 (d, *J* = 8.5 Hz, 2H); 7.38 (d, *J* = 1.8 Hz, 4H); 6.68 (d, *J* = 8.5 Hz, 2H); 4.71–4.62 (m, 2H); 4.31 (dd, *J*_1_ = 8.2 Hz, *J*_2_ = 4.6 Hz, 1H); 4.06 (t, *J* = 7.2 Hz, 4H); 3.86 (s, 4H); 3.12 (s, 3H); 2.35–1.96 (m, 4H); 1.781.63 (m, 4H); 1.14–0.91 (m, 16H).

^13^C NMR (100.63 MHz, D_2_O) *δ* (ppm): 181.66; 178.61; 168.10; 162.50; 160.74; 151.03; 148.68; 135.57; 128.60; 123.45; 123.41; 122.05; 122.00; 121.87; 120.37; 111.34; 55.55; 49.90; 49.37; 39.00; 35.61; 35.58; 34.02; 29.12; 28.90; 28.64; 28.54; 28.09; 25.32.

IR (ATR) *ν*_max_ (cm^−1^): 3319, 3153, 2924, 2850, 1585, 1556, 1507, 1449, 1380, 1208, 1168, 1097, 826.

Elemental analysis calcd for C_40_H_56_N_12_O_5_·8.5H_2_O (%): C 51.21; H 7.84; N 17.92; found: C 51.07; H 7.43; N 18.14.

###### [C_12_MIM]_2_[MTX]

Following the general procedure, [C_12_MIM][OH] (146 mg; 0.44 mmol) was added to a methotrexate solution. The final product was obtained as a yellow wax (203 mg; 97%).

^1^H NMR (400.13 MHz, D_2_O) *δ* (ppm): 8.33 (s, 1H); 7.62 (d, *J* = 6.80 Hz, 2H); 7.30 (d, *J* = 1.60 Hz, 2H); 7.16 (d, *J* = 1.60 Hz, 2H); 6.59 (d, *J* = 6.80 Hz, 2H); 4.57 (s, 1H); 4.23 (t, *J* = 5.20 Hz, 1H); 3.74 (s, 6H); 3.0 (s, 3H); 2.14–1.88 (m, 4H); 1.46 (s br, 4H); 1.16–0.92 (m, 38H); 0.76 (t, *J* = 5.60 Hz, 3H).

^13^C NMR (100.63 MHz, D_2_O) *δ* (ppm): 181.46; 178.24; 167.0; 162.68; 162.22; 154.05; 150.78; 148.79; 146.92; 135.83; 135.58; 135.32; 128.76; 123.64; 121.91; 121.71; 121.59; 111.43; 55.54; 55.17; 49.19; 38.96; 35.73; 34.20; 31.98; 29.84; 29.82; 29.78; 29.59; 29.54; 29.49; 29.04; 26.03; 22.64; 13.89.

IR (ATR) *ν*_max_ (cm^−1^): 3319, 2924, 2850, 1628, 1553, 1504, 1445, 1380, 1204, 1168, 1097, 826.

Elemental analysis calcd for C_52_H_94_N_12_O_11_·6H_2_O (%): C 58.73; H 8.91; N 15.81; found: C 58.56; H 9.07; N 15.85.

###### [C_2_OHMIM]_2_[MTX]

Following the general procedure, [C_2_OHMIM][OH] (71 mg; 0.44 mmol) was added to a methotrexate solution. The final product was obtained as a yellow wax (136 mg; 87%).

^1^H NMR (400.13 MHz, D_2_O) *δ* (ppm): 8.52 (s, 1H); 7.65 (d, *J* = 6.80 Hz, 2H); 7.45 (d, *J* = 1.60 Hz, 2H); 7.40 (d, *J* = 1.60 Hz, 2H); 6.8 (d, *J* = 7.20 Hz, 2H); 4.69 (s, 2H); 4.27 (t, *J* = 4.0 Hz, 4H); 3.90 (t, *J* = 3.20 Hz, 4H) 3.86 (s, 6H); 3.13 (s, 3H); 2.31–2.23 (m, 2H); 2.16–2.09 (m, 1H); 2.04–1.96 (m, 1H).

^13^C NMR (100.63 MHz, D_2_O) *δ* (ppm): 182.18; 179.02; 168.94; 162.72; 161.94; 153.32; 151.48; 149.16; 148.04; 136.03; 128.74; 123.48; 122.31; 122.02; 120.43; 111.63; 59.69; 55.82; 54.71; 51.46; 38.83; 35.62; 34.31; 28.67.

IR (ATR) *ν*_max_ (cm^−1^): 3312, 3211, 1582, 1556, 1511, 1449, 1380, 1204, 1162, 1067, 826.

Elemental analysis calcd for C_52_H_94_N_12_O_11_·8.5H_2_O (%): C 44.70; H 6.92; N 19.55; found: C 44.81; H 7.07; N 19.59.

###### [C_10_MIM]_2_[MTX]

Following the general procedure, [C_10_MIM][OH] (133.4 mg; 0.44 mmol) was added to a methotrexate solution. The final product was obtained as a yellow wax (172 mg; 88%).

^1^H NMR (400.13 MHz, D_2_O) *δ* (ppm): 8.49 (s, 1H); 7.68 (d, *J* = 6.80 Hz, 2H); 7.39 (d, *J* = 1.60 Hz, 2H); 7.32 (d, *J* = 1.60 Hz, 2H); 6.73 (d, *J* = 6.80 Hz, 2H); 4.68 (s, 2H); 4.30 (q, *J*_1_ = 6.80 Hz, *J*_2_ = 4.0 Hz, 1H); 4.02 (t, *J* = 6.0 Hz, 4H); 3.84 (s, 6H); 3.10 (s, 3H); 2.28–2.17 (m, 2H); 2.14–2.07 (m, 1H); 2.03–1.95 (m, 1H); 1.64 (s br, 4H) 1.18–1.04 (m, 30H); 0.78 (t, *J* = 5.6 Hz, 6H).

^13^C NMR (100.63 MHz, D_2_O) *δ* (ppm): 181.67; 178.43; 167.54; 162.79; 162.24; 153.95; 151.10; 149.01; 147.31; 128.78; 123.61; 122.03; 121.89; 121.31; 111.50; 55.61; 49.32; 38.81; 35.71; 34.27; 31.67; 29.43; 29.26; 29.12; 29.05; 28.65; 25.76; 22.42; 13.77.

IR (ATR) *ν*_max_ (cm^−1^): 3325, 3153, 2924, 2850, 1628, 1556, 1504, 1445, 1377, 1204, 1168, 1094, 826, 761.

Elemental analysis calcd for C_48_H_74_N_12_O_5_·6.5H_2_O(%): C 56.73; H 8.63; N 16.54; found: C 56.89; H 8.64; N 17.03.

###### [C_10_-3-Pic]_2_[MTX]

Following the general procedure, [C_10_-3-Pic][OH] (13 8 mg; 0.44 mmol) was added to a methotrexate solution. The final product was obtained as a yellow wax (183 mg; 90%).

^1^H NMR (400.13 MHz, D_2_O) *δ* (ppm): 8.57 (s, 2H); 8.53 (d, *J* = 4.80 Hz, 2H); 8.46 (s, 1H); 8.27 (d, *J* = 6.40 Hz, 2H); 7.86 (t, *J* = 5.20 Hz, 2H); 7.65 (d, *J* = 6.80 Hz, 2H); 4.65 (s, 2H); 4.42 (t. *J* = 6.0 Hz, 4H); 4.31 (q, *J*_1_ = 6.40 Hz, *J*_2_ = 4H.0z, 1H); 3.07 (s, 3H); 2.45 (s, 6H); 2.28–2.17 (m, 2H); 2.14–2.07 (m, 1H); 2.03–1.96 (m, 1H); 1.80–1.74 (m, 4H); 1.11–0.97 (m, 30H); 0.70 (t, *J* = 6.0 Hz, 6H).

^13^C NMR (100.63 MHz, DMSO-*d*_6_) *δ* (ppm): 181.67; 178.43; 167.54; 162.79; 162.24; 153.95; 151.00; 149.01; 147.31; 128.78; 123.61; 122.03; 121.89; 121.31; 111.50; 55.61; 49.32; 38.81; 35.71; 34.27; 31.67; 29.43; 29,26; 29.12; 29.05; 28.65; 25.76; 22.42; 13.77.

IR (ATR) *ν*_max_ (cm^−1^): 3332, 3198, 2924, 2853, 1625, 1585, 1556, 1504, 1445, 1380, 1204, 1094, 826.

Elemental analysis calcd for C_52_H_76_N_10_O_5_·6H_2_O (%): C 60.68; H 8.62; N 13.61; found: C 60.40; H 8.51; N 13.29.

###### [C_2_OH-3-Pic]_2_[MTX]

Following the general procedure, [C_2_OH-3-Pic][OH] (96 mg; 0.44 mmol) was added to a methotrexate solution. The final product was obtained as a yellow wax (156 mg; 98%).

^1^H NMR (400.13 MHz, D_2_O) *δ* (ppm): 8.57 (s, 2H); 8.54 (d, *J* = 6.0 Hz, 2H); 8.45 (s, 1H); 8.23 (d, *J* = 8.0 Hz, 2H); 7.82 (t, *J* = 7.20 Hz, 2H); 7.58 (d, *J* = 6.80 Hz, 2H); 6.68 (d, *J* = 9.20 Hz, 2H); 4.60–4.57 (m, 6H); 4.27 (q, *J*_1_ = 8.40 Hz, *J*_2_ = 4.80 Hz, 1H); 4.01 (t, *J* = 5.20 Hz, 4H); 3,08 (s, 3H); 2.44 (s, 6H); 2.30–2.19 (m, 2H); 2.16–2.07 (m, 1H); 2.04–1.94 (m, 1H).

^13^C NMR (100.63 MHz, D_2_O) *δ* (ppm): 182.11; 178.94; 168.79; 162.61; 161.85; 153.23; 151.40; 149.21; 148.01; 146.17; 143.88; 141.57; 139.73; 128.73; 127.18; 121.91; 120.35; 111.62; 63.24; 60.28; 55.77; 54.59; 38.94; 34.27; 28.66; 17.56.

IR (ATR) *ν*_max_ (cm^−1^): 3325, 3208, 1585, 1553, 1504, 1445, 1377, 1204, 1074, 761.

Elemental analysis calcd for C_36_H_44_N_10_O_7_·7H_2_O (%): C 50.58; H 6.84; N 16.38; found: C 50.93; H 6.82; N 15.85.

###### [C_2_MIM]_2_[MTX]

Following the general procedure, [C_2_MIM][OH] (84 mg; 0.44 mmol) was added to a methotrexate solution. The final product was obtained as a yellow wax (137 mg; 93%).

^1^H NMR (400.13 MHz, D_2_O) *δ* (ppm): 8.63 (s, 2H); 8.53 (s, 1H); 7.65 (d, *J* = 8.80 Hz, 2H); 7.37 (d, *J* = 22.40 Hz, 4H); 6.8 (d, *J* = 8.80 Hz, 2H); 4.69 (s, 2H); 4.28 (q, *J*_1_ = 8.80 Hz, *J*_2_ = 4.40 Hz, 1H); 4.15 (q, *J*_1_ = 15.20, *J*_2_ = 7.20 Hz, 4H); 3.83 (s, 6H); 3.13 (s, 3H); 2.33–2.21 (m, 2H); 2.18–2.08 (m, 1H); 2.04–1.94 (m, 1H); 1.44 (t, *J* = 7.20 Hz, 6H).

^13^C NMR (100.63 MHz, D_2_O) *δ* (ppm): 182.16; 178.99; 168.88; 162.72; 161.58; 152.81; 151.46; 149.18; 148.28; 135.38; 128.71; 123.33; 121.99; 121.72; 120.41; 111.61; 55.78; 54.68; 44.68; 44.66; 38.84; 35.50; 35.47; 34.26; 28.64; 14.35.

IR (ATR) *ν*_max_ (cm^−1^): 3325, 1585, 1556, 1507, 1445, 1383, 1208, 1168, 1097, 826.

Elemental analysis calcd for C_32_H_42_N_12_O_5_·6.5H_2_O (%): C 48.54; H 7.0; N 21.23; found: C 48.48; H 6.73; N 20.75.

###### [C_6_MIM]_2_[MTX]

Following the general procedure, [C_6_MIM][OH] (109 mg; 0.44 mmol) was added to a methotrexate solution. The final product was obtained as a yellow wax (182 mg; 87%).

^1^H NMR (400.13 MHz, D_2_O) *δ* (ppm): 8.63 (s, 1H); 7.73 (d, *J* = 8.80 Hz, 2H); 7.42 (d, *J* = 8.0 Hz, 4H); 6.92 (d, *J* = 8.80 Hz, 2H); 4.31 (q, *J*_1_ = 8.40 Hz, *J*_2_ = 4.40 Hz, 1H); 4.14 (t, *J* = 6.80 Hz, 4H); 3.88 (s, 6H); 3.201 (s, 3H); 2.34–2.24 (m, 2H); 2.19–2.11 (m, 1H); 2.06–1.97 (m, 1H); 1.85–1.78 (m, 4H); 1.28–1.21 (m, 12H); 0.82 (t, *J* = 6.40 Hz, 6H).

^13^C NMR (100.63 MHz, D_2_O) *δ* (ppm): 182.24; 179.06; 169.23; 163.13; 162.18; 153.57; 151.79; 149.43; 148.34; 128.85; 123.37; 122.34; 122.05; 120.71; 111.99; 55.80; 54.94; 49.44; 38.89; 35.52; 34.28; 30.25; 29.04; 28.60; 24.91; 21.70; 13.14.

IR (ATR) *ν*_max_ (cm^−1^): 3322, 3153, 2924, 1625, 1585, 1553, 1445, 1380, 1208, 1168, 1097, 830, 764.

Elemental analysis calcd for C_40_H_58_N_12_O_5_·9H_2_O (%): C 50.62; H 8.07; N 17.71; found: C 50.81; H 7.24; N 16.68.

###### [C_8_MIM]_2_[MTX]

Following the general procedure, [C_8_MIM][OH] (101 mg; 0.44 mmol) was added to a methotrexate solution. The final product was obtained as a yellow wax (196 mg; 91%).

^1^H NMR (400.13 MHz, D_2_O) *δ* (ppm): 8.62 (s, 1H); 7.71 (d, *J* = 8.40 Hz, 2H); 7.41 (d, *J* = 10.40 Hz, 4H); 6.91 (d, *J* = 8.400 Hz, 2H); 4.29 (q, *J*_1_ = 9.20 Hz, *J*_2_ = 4.0 Hz, 1H); 4.13 (t, *J* = 7.20 Hz, 4H); 3.86 (s, 6H); 3.20 (s, 3H); 2.29–2.22 (m, 2H); 2.18–2.08 (m, 1H); 2.03–1.94 (m, 1H); 1.83–1.75 (m, 4H); 1.27–1.13 (m, 22H); 0.82 (t, *J* = 6.40 Hz, 6H).

^13^C NMR (100.63 MHz, D_2_O) *δ* (ppm): 182.12; 178.94; 168.94; 163.01; 162.14; 153.53; 151.60; 149.39; 148.27; 128.90; 123.39; 122.26; 122.04; 120.58; 111.90; 55.71; 54.85; 49.43; 39.02; 35.54; 34.25; 30.92; 29.05; 28.72; 28.14; 27.93; 25.21; 21.93; 13.34.

IR (ATR) *ν*_max_ (cm^−1^): 3315, 3149, 2924, 2856, 1631, 1553, 1507, 1445, 1377, 1208, 1168, 1097, 826.

Elemental analysis calcd for C_44_H_66_N_12_O_5_·7.5H_2_O (%): C 54.03; H 8.35; N 17.18; found: C 54.14; H 7.44; N 16.35.

### 2.3 MTX-salt characterization

#### NMR spectra

^1^H NMR and ^13^C NMR spectra in D_2_O (from Eurisotop) were recorded on a Bruker AMX 400 spectrometer at room temperature unless specified otherwise.

To perform NMR, 5 mm borosilicate tubes were used, and the sample concentration was approximately 7 mg mL^−1^ for ^1^H NMR and 30 mg mL^−1^ for ^13^C NMR. Chemical shifts are reported in parts per million (ppm).

#### Elemental analysis

The elemental analysis experiments were performed in a Thermo-Finnigan-CE Instruments Flash EA 1112 CHNS series under standard conditions (T combustion reactor 900°C T GC column furnace 65°C, multiseparation SS GC column, He flow 130 mL min^−1^, O_2_ flow 250 mL min^−1^).

#### Infrared spectra

Infrared (IR) spectra were acquired using a Perkin-Elmer Spectrum-Two FTIR spectrophotometer equipped with a UATR module. Transmittance of the samples was recorded between 4000 and 400 cm^−1^.

### 2.4 Biological studies

#### Cell lines and maintenance

Primary dermal neonatal fibroblasts (PCS-201-010) and lung adenocarcinoma cell lines (A549 and H1975) were acquired from ATCC (Manassas, VA, USA). Fibroblasts, A549 and H1975 cells were cultured in Dulbecco's modified Eagle medium (DMEM, Thermo Fisher Scientific) according to the manufacturer's instructions and previously described procedures.^26^ Media were supplemented with 10% (v/v) fetal bovine serum (FBS, Thermo Fisher Scientific) and a mixture of 100 U mL^−1^ penicillin and 100 mg mL^−1^ streptomycin. All experiments were conducted at 37 °C, 5% (v/v) CO_2_, and 99% humidity in the dark. For simplicity, the supplemented media will be referred from now on as DMEM. NSCLC cell lines used in this work represent two different cancer models with different driver mutations^27^ (Table 1).

**Table 1:** Driver mutations in the NSCLC cell lines used in this study

Cell line	Gene	Driver mutation
A549	*KRAS*	p.G12S
H1975	*EGFR*	p.T790M, p.L858R

#### Antiproliferative activity

Cells were seeded in a 96-well plate at a density of 7500 cells per well for 24 h and after the medium was substituted by fresh medium in the absence or presence of different concentrations of MTX-salts (0.1–50 μM range). Doxorubicin (1.5 μM) was used as a positive control and 0.1% (v/v) DMSO was used as a vector control in all biological replicates. After 48 h of incubation, cell viability was measured using the Cell Titer 96® Aqueous One solution cell proliferation assay (Promega, Madison, WI, USA) according to the manufacturer's instructions and procedures described previously.^26,28^ The absorbance of the formazan produced was quantified with a Tecan microplate reader (Infinite M200 (Tecan, Mannedorf, Switzerland)) and analysis of the dose–response curves to determine the relative IC_50_ was performed with GraphPad Prism 8 software (GraphPad Software, La Jolla, CA, USA). The selectivity index (IC_50_ fibroblasts/IC_50_ lung cancer cell line) was used as a measurement of the specificity of MTX salts for lung cancer cells.

#### Evaluation of apoptosis

A549 cells were seeded on 6-well plates at a density of 2 × 10^5^ cells per well and incubated for 48 h with the IC_50_ of salts ([C_12_mim]_2_[MTX], [C_10_mim]_2_[MTX], and [C_10_-3-picoline]_2_[MTX]) previously determined. Doxorubicin (1.5 μM) was used as a positive control and 0.1% (v/v) DMSO as a vector control. Cells were then detached from the wells and double stained using dead cell apoptosis annexin V (Thermo Fisher Scientific) according to the manufacturer's instructions. The percentage of live cells (unstained), cells in initial apoptosis (stained with annexin V-Alexa Fluor 488), cells in late apoptosis (double stained with annexin V-Alexa Fluor 488 and propidium iodide), or cells in necrosis (stained with propidium iodide) were quantified using an Attune acoustic focusing flow cytometer and the respective software (Thermo Fisher Scientific).

#### Production of reactive oxygen species

The production of ROS was evaluated using the dye 2′,7′-dichlorodihydrofluorescein diacetate (H_2_DCF-DA, Thermo Fisher Scientific) according to previously described procedures.^26,28^ A549 cells were seeded on 6-well plates at a cell density of 2 × 10^5^ cells per well and incubated for 48 h with the IC_50_ of the ILs as previously mentioned, 0.1% (v/v) DMSO as a vector control, 100 μM *tert*-butyl hydroperoxide (TBHP) or 1.5 μM doxorubicin (positive controls). The fluorescence was quantified in an Attune acoustic focusing cytometer with the respective software (Thermo Fisher Scientific) and normalized to the fluorescence of vector control sample.

#### Mitochondrial membrane potential

The mitochondrial potential was quantified using a JC-1 mitochondrial membrane potential assay kit (Abnova, Taipé, Taiwan) according to the manufacturer's instructions and as described previously.^26,28^ Briefly, 2 × 10^5^ cells per well A549 cells were seeded on each well of a 6-well plate and incubated for 48 h with the IC_50_ of ILs as previously mentioned, 0.1% (v/v) DMSO as a vector control and 1.5 μM doxorubicin. Cells with red (JC-1 aggregated) and green (JC-1 monomer) fluorescence were quantified with an Attune acoustic focusing flow cytometer and the respective software (Thermo Fisher Scientific), and fluorescence ratios were normalized to control samples incubated with DMSO.

#### Statistical analysis

All data concerning cell-based assays were expressed as mean ± SEM from at least three independent experiments. Statistical significance was evaluated using the Student's *t* test; *p* < 0.05 was considered statistically significant. Statistical analysis was performed using GraphPad Prism v8.01 (GraphPad Software, La Jolla, CA, USA).

## 3. Results and discussion

### 3.1 Synthesis and characterization of MTX-salts

**Fig. 2:**
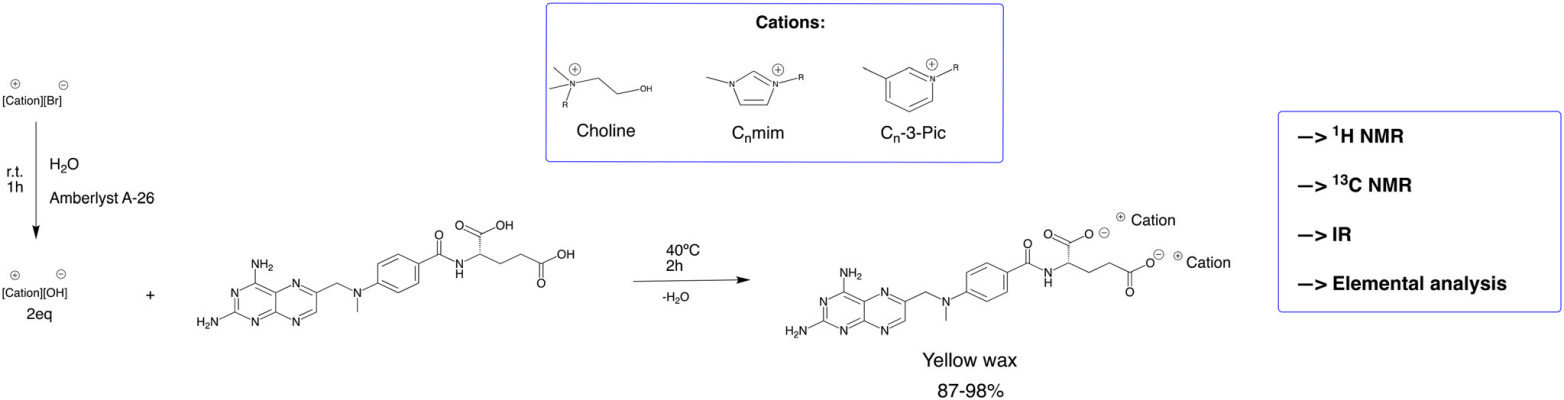
The synthetic methodology followed for the preparation of MTX salts.

MTX salts were synthesized, in the first step, by an exchange reaction from bromide or chloride to hydroxide using the resin Amberlyst A-26 in an aqueous solution. Then, MTX (1.0 molar equivalent) was dissolved in water at 40 °C, and the desired counter ion (2.0 molar equivalents) was added. After 2 h without the presence of light, the solvent was removed under reduced pressure to obtain the desired product. The product was dried under vacuum for 24 h. MTX possesses two acidic protons (p*K*_a_ = 2.9 and 4.6)^29^ in the carboxylic acids that can be deprotonated under suitable conditions. When the counter ions were treated with Amberlyst A-26, the pH of the solution was monitored to confirm adequate basicity to deprotonate the corresponding two acidic protons. Additionally, MTX salts were then dissolved in cellular medium DMEM (pH 7–7.6), which indicates that MTX seems to be deprotonated in DMEM solution. Fig. 2 illustrates the synthetic methodology for the preparation of different MTX salts.

Choline, imidazolium, and picolinium derivatives were selected as cations for combination with the MTX di-anion, based on our previous knowledge of their biocompatibility and their effectiveness in modulating APIs properties like solubility or cytotoxicity.^17,18,23^ The similarities between counter ions and the different alkyl chain lengths are important parameters for understanding structure–activity correlation.

**Fig. 3:**
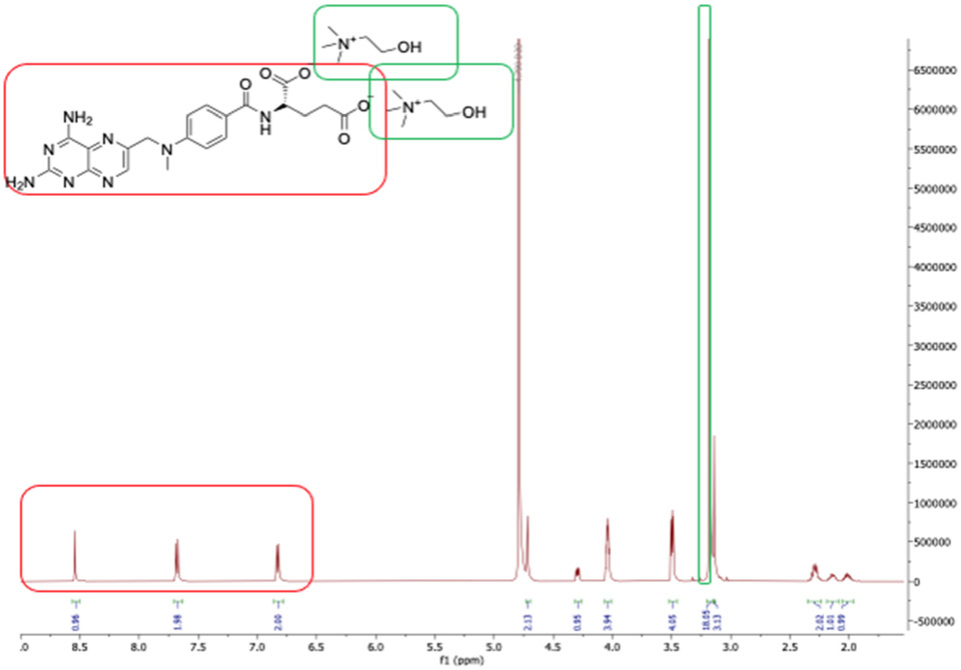
^1^H NMR spectra of [choline]_2_[MTX].

MTX salts were characterized by ^1^H NMR, ^13^C NMR, and elemental analysis. ^1^H NMR allowed us to elucidate the cation–anion ratio (2 : 1 between counter ions and MTX) as well as the chemical stability of the desired product. In Fig. 3, the red boxes show the aromatic protons of MTX while the green boxes illustrate the methyl groups of choline (which by proton integration is proof of the 2 : 1 cation–anion ratio, as expected).

### 3.2 Physicochemical properties

**Fig. 4:**
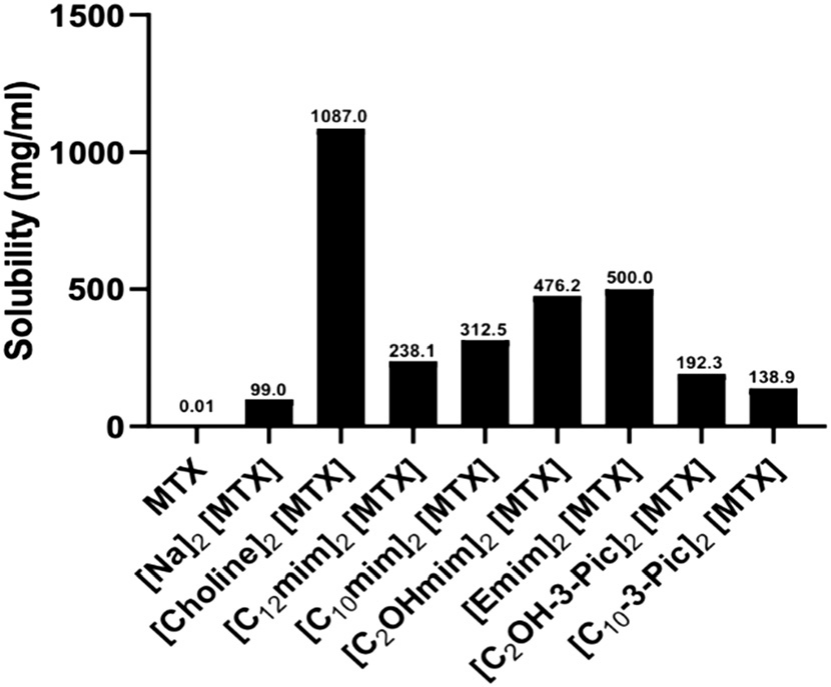
Solubility of MTX salts in water at room temperature.

To study the solubility and stability of the MTX salts, two different assays were performed. To measure the solubility, 1 μL of water was added dropwise to 1 mg of MTX salt and the solubility was visually determined. Fig. 4 shows the results for the solubility of MTX salts in water. [Choline]_2_[MTX], as expected, increased 10-fold compared to original [Na]_2_[MTX]. The remaining MTX-salts also showed some improvement in solubility (between 1.3- and 5-fold) compared to original MTX and [Na]_2_[MTX].

As biological assays were performed for a 48 h period of incubation in the presence of MTX and MTX salts, a UV-vis spectroscopy assay was performed to confirm that, despite its reduced solubility in aqueous-based solutions, MTX stability was retained in DMEM biological medium during the biological assays.

### 3.3 Biological assays

#### 3.3.1 Antiproliferative activity and SAR studies

Due to the anticancer properties of MTX, in particular, against lung cancer cells,^4,5^ studies of the antiproliferative effects of MTX salts were performed on two lung adenocarcinoma cancer cell lines (A549 and H1975) both isolated from lung cancer patients with different driver mutations (Table 1). Moreover, to understand the cytotoxic effect of MTX salts in normal cells, MTX salts and counter ions were additionally tested in a normal primary human dermal fibroblast and the IC_50_ values from all cell lines are presented in Table 2.

**Table 2:** IC_50_ values of MTX salts and counter-ions in NSCLC cancer cell lines, A549 and H1975, and in normal fibroblasts

MTX-salts	IC_50_ (μM) A549	IC_50_ (μM) H1975	IC_50_ (μM) fibroblasts	SI (A549)	SI (H1975)
MTX	>50	n.p.	n.p.	—	—
[Na]_2_[MTX]	3.82 ± 0.58	>50	>50	—	—
[Choline]_2_[MTX]	>50	n.p.	n.p.	—	—
[N_10,1,1,2OH_]_2_[MTX]	19.15 ± 1.28	n.p.	n.p.	—	—
[N_12,1,1,2OH_]_2_[MTX]	8.55 ± 0.93	n.p.	n.p.	—	—
[C_2_MIM]_2_[MTX]	>50	n.p.	n.p.	—	—
[C_2_OHmim]_2_[MTX]	>50	n.p.	n.p.	—	—
[C_6_mim]_2_[MTX]	>50	n.p.	n.p.	—	—
[C_8_mim]_2_[MTX]	>50	n.p.	n.p.	—	—
[C_10_mim]_2_[MTX]	1.71 ± 0.23	14.90 ± 1.17	7.61 ± 0.88	4.5	0.5
[C_12_mim]_2_[MTX]	0.55 ± 0.25	2.72 ± 0.43	1.59 m ± 0.20	2.9	0.6
[C_2_OH-3-picoline]_2_[MTX]	>50	n.p.	n.p.	—	—
[C_10_-3-picoline]_2_[MTX]	0.94 ± 0.03	29.12 ± 1.46	10.62 ± 1.03	11.3	0.4
[MIMC_12_MIM][MTX]	12.38 ± 1.09	n.p.	n.p.	—	—
[Choline][Cl]	>50	n.p.	n.p.	—	—
[N_10,1,1,2OH_][Br]	n.p.	n.p.	n.p.	—	—
[N_12,1,1,2OH_][Br]	n.p.	n.p.	n.p.	—	—
[C_2_MIM][Br]	>50	n.p.	n.p.	—	—
[C_2_OHmim][Br]	>50	n.p.	n.p.	—	—
[C_6_mim][Br]	n.p.	n.p.	n.p.	—	—
[C_8_mim][Br]	n.p.	n.p.	n.p.	—	—
[C_10_mim][Br]	1.1 ± 0.02	19.25 ± 1.28	>50	45.5	2.6
[C_12_mim][Br]	0.60 ± 0.21	10.22 ± 1.01	8.30 ± 0.92	13.83	0.8
[C_2_OH 3-picoline][Br]	>50	n.p.	n.p.	—	—
[C_10_-3-picoline][Br]	1.03 ± 0.01	29.30 ± 1.47	>50	48.5	1.7
[MIMC_12_MIM][Br]	n.p.	n.p.	n.p.	—	—

**Fig. 5:**
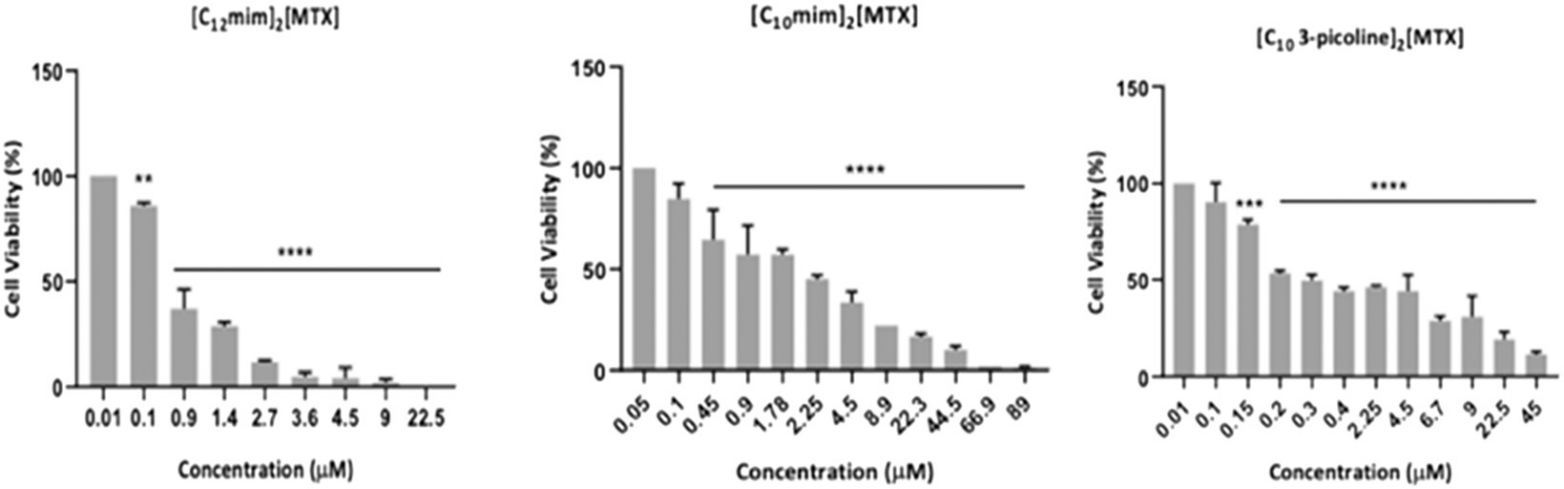
Viability of A549 cancer cell line after exposure to different concentrations of [C_12_mim]_2_[MTX], [C_10_-3-picoline]_2_[MTX] and [C_10_mim]_2_[MTX] for 48 h. DMSO was used as the vehicle control. Data are expressed as the mean ± SEM of three biological assays. **p value < 0.01 ***p value < 0.005, ****p value < 0.0001.

The following eight MTX salts, [Na]_2_[MTX], [choline]_2_[MTX], [C_2_MIM]_2_[MTX], [C_2_OHmim]_2_[MTX], [C_10_mim]_2_[MTX], [C_12_mim]_2_[MTX], [C_2_OH-3-picoline]_2_[MTX], [C_10_-3-picoline]_2_[MTX], were synthesized and characterized, and their antiproliferative profiles using the A549 cell line were evaluated. (see Table 2 and Fig. S27 to S33†). In general, [C_12_mim]_2_[MTX], [C_10_mim]_2_[MTX], and [C_10_-3-picoline]_2_[MTX] showed the highest antiproliferative activity in A549 (Table 2 and Fig. 5). The order of cytotoxicity is [C_12_mim]_2_[MTX] (IC_50_ = 0.55 ± 0.25) > [C_10_-3-picoline]_2_[MTX] (IC_50_ = 0.94 ± 0.03) > [C_10_mim]_2_[MTX] (IC_50_ = 1.71 ± 0.23).

Then, these selected MTX salts were tested in H1975 (Table 2 and Fig. S34 to S39†). As can be observed in Table 2, the cytotoxic effects of [C_12_mim]_2_[MTX], [C_10_mim]_2_[MTX], and [C_10_-3-picoline]_2_[MTX] were higher in A549 than those in H1975. This is an interesting result, as mutated EGFR seems to confer resistance to these MTX-salts, while KRAS codon 12 mutation seems to increase sensitivity. Indeed, RAS alterations are the most common activating lesions in human cancers, and KRAS is the most common oncogene-driven form of NSCLC, accounting for more than one-quarter of patients.^30^

Moreover, [C_12_mim]_2_[MTX], [C_10_mim]_2_[MTX] and [C_10_-3-picoline]_2_[MTX] showed a lower IC_50_ in A549 than [Na]_2_[MTX] (3.82 ± 0.58), which means that they are more cytotoxic compared to sodium MTX as a reference. Additionally, in most cases the bromide salts exhibited higher IC_50_ values than the respective MTX-counter ion salts in both cancer cell lines:

[C_12_mim][Br] (IC_50_ = 0.60 ± 0.21); [C_10_-3-picoline][Br] (IC_50_ = 1.03 ± 0.01); [C_10_mim][Br] (IC_50_ = 1.1 ± 0.02) in A549 and [C_12_mim][Br] (IC_50_ = 10.22 ± 1.01); [C_10_-3-picoline][Br] (IC_50_ = 29.30 ± 1.47); [C_10_mim][Br] (IC_50_ = 19.25 ± 1.28) in H1975.

These results showed that the synergetic effect between MTX and counter ions improves the solubility profile as well as increases its antiproliferative activity of the MTX salts.

Interestingly, these three MTX salts showed higher IC_50_ in fibroblasts, indicating their specificity to A549 NSCLC cells (Table 2). In particular, [C_10_-3-picoline]_2_[MTX] showed an IC_50_ in the cancer cell line 4.06-fold higher than that in [Na]_2_[MTX] and, in addition, it possesses a selectivity index (SI) of 11.3 (IC_50_ fibroblast/IC_50_ A549) (Table 2). The SI measures the specific cytotoxicity of a compound in a cancer cell line compared to that in normal cells (fibroblasts). The higher SI value means higher specificity of compounds to cancer cells.

Once again, a higher SI value was observed for A549 compared to that of H1975 NSCLC cells (Table 2), which indicating a higher therapeutic index for A549 cells in the case of these MTX salts. [C_10_-3-picoline]_2_[MTX] possesses a higher SI and it was considered the most promising for therapeutic applications.

Concerning the structure–activity relationship, the results showed that longer alkyl chains improve the antiproliferative effect when linked to an imidazole or picoline ring. Conversely, longer alkyl chains linked to choline do not change the original antiproliferative activity. Other counter ions such as [C_6_mim], [C_8_mim], [mimC_12_mim], or [C_2_OH-3-picoline] showed no significant improvement in the antiproliferative effect in A549 (Table 2). This observation can be attributed to the presence of shorter alkyl chain moieties.

Compared to other MTX salts reported in the literature, our MTX salts show higher antiproliferative activity.^15^ For instance, [TBP][MTX], [ProEt][MTX] and [AspEt][MTX]^15^ show an IC_50_ of around 50 μM in B16F10 cells, which indicates that the counter ions used in this work and the approach with two equivalents are more suitable. Nevertheless, [ProEt][MTX] demonstrated protection from chemical and enzymatic degradation, which improved the oral bioavailability.^15^ The authors demonstrated that [ProEt][MTX] has reduced side effects and better *in vivo* antitumor activity compared to that of [Na][MTX],^15^ using biodistribution studies.^15^

**Fig. 6:**
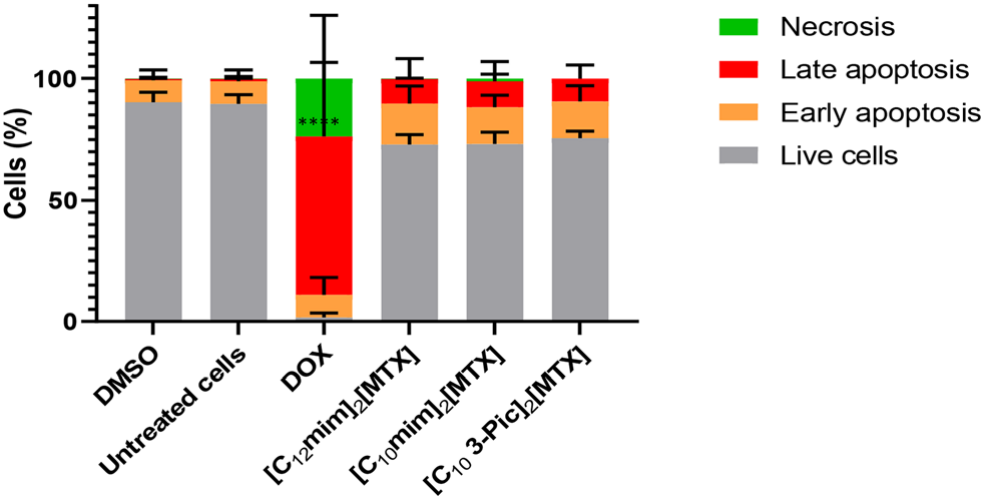
Evaluation by flow cytometry using annexin V/PI double staining of live (grey), early apoptotic (orange), late apoptotic (red) and necrotic (green) A549 cells after 48 h of exposure to IC_50_ concentrations of compounds. 0.1% (v/v) (DMSO was used as vehicle control, and DOX (1.5 μM), as a positive control. The results presented are the average ± SD of three independent assays). ****p value < 0.0001.

For a more complete characterization of the antiproliferative effect for the three most promising MTX-salts ([C_12_mim]_2_[MTX], [C_10_mim]_2_[MTX], and [C_10_-3-picoline]_2_[MTX]), cell death by necrosis and apoptosis were evaluated (Fig. 6).

#### 3.3.2 Apoptosis evaluation

Cellular apoptosis is a process that leads to cell death. Using annexin V and propidium iodide (PI), cells could be divided into live cells, early apoptotic cells, late apoptotic cells, or necrotic cells, based on the differences in plasma membrane of these different stages. PI only enters cells with a permeabilized and/or compromised membrane (late apoptotic and necrotic cells, respectively), while annexin V-FITC binds to phosphatidylserine that translocates from the inner side of the plasma membrane to the outer side, staining cells in initial/late apoptosis.^31^ As indicated in Fig. 6 and Table 3, after 48 h of incubation in the presence of the three MTX salts, apoptosis (early and late) increased approximately 15% (from 10 to 25%) compared to the control cells (DMSO), despite not being statistically significant.

**Table 3:** Percentage of live cells, apoptotic, and necrotic A549 cells after 48 h of exposure to IC_50_ of [C_12_mim]_2_[MTX], [C_10_mim]_2_[MTX], [C_10_-3-picoline]_2_[MTX] and the controls DMSO, untreated cells and DOX

Compound	Live cells	Early apoptosis	Late apoptosis	Necrosis
DMSO	90.2 ± 4.1	9.0 ± 4.2	0.6 ± 1.0	0.06 ± 0.07
Untreated cells	89.5 ± 3.8	9.3 ± 4.6	1.0 ± 0.5	0.06 ± 0.07
DOX	1.7 ± 1.8	9.3 ± 7.1	65.25 ± 30.4	23.7 ± 26.0
[C_12_mim]_2_ [MTX]	72.9 ± 4.1	16.7 ± 7.3	10.2 ± 8.3	0.1 ± 0.2
[C_10_mim]_2_ [MTX]	73.2 ± 5.0	14.9 ± 5.0	10.7 ± 8.1	1.1 ± 1.8
[C_10_-3-picoline]_2_ [MTX]	75.3 ± 3.0	14.9 ± 6.5	9.4 ± 5.6	0.02 ± 0.02

Moreover, almost no necrosis is observed in the presence of MTX-salts, contrary to the positive control (doxorubicin) (Table 3 and Fig. 6). This is a positive result because necrotic cells release content that includes molecules acting as signals to promote inflammation, which should be avoided in a cancer context.^32^

**Fig. 7:**
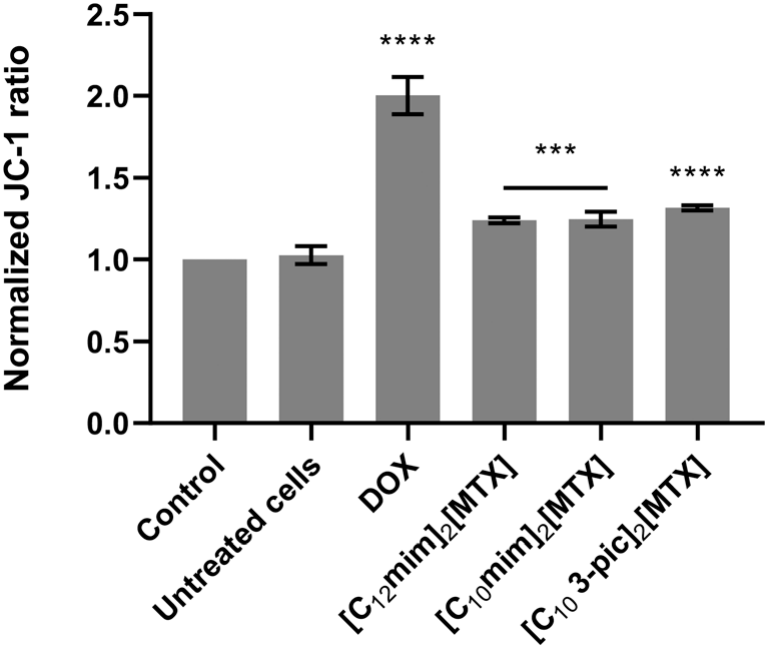
Evaluation of alterations in the mitochondrial membrane potential of A549 cells exposed for 48 h to 0.1% (v/v) DMSO, as the vehicle control, or to DOX (1.5 μM), as a positive control, and compounds at the respective IC_50_ concentrations (the results presented are the average ± SD of three independent assays. ***p value < 0.005, ****p value < 0.0001).

To elucidate the mechanisms underlying apoptotic cell death, the mitochondrial membrane potential (Fig. 7) was also evaluated.

#### 3.3.3 Mitochondrial membrane potential

As mitochondria are involved in the apoptosis, the mitochondrial membrane potential is compromised (Fig. 7). JC-1 is a cationic dye that naturally enters the mitochondria and accumulates in the form of JC-1 aggregates that exhibit red fluorescence. Once the mitochondrial membrane potential is compromised (depolarization), the dye is no longer retained inside the mitochondria and is found in the cytosol in its monomeric form, exhibiting a green fluorescence. The ratio between red and green fluorescence could give some information about polarization of mitochondria. In this case, regarding the three MTX salts, JC-1 normalization is slightly higher than one, which means that JC-1 is predominantly inside the mitochondria due to the hyperpolarization of the membrane. Hyperpolarization occurs in the early stages of apoptosis, which matches the apoptosis results^33^ and indicates that the apoptosis is probably intrinsic (*via* mitochondria).

#### 3.3.4 Production of reactive oxygen species

**Fig. 8:**
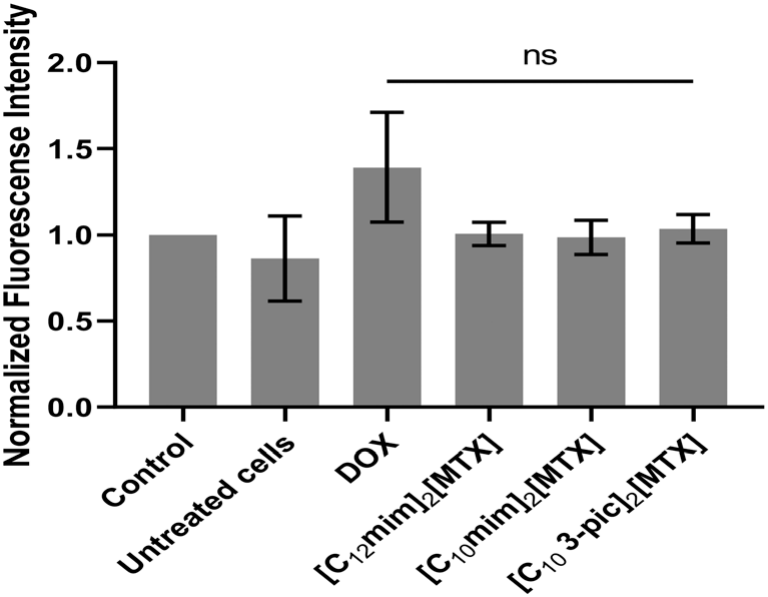
Evaluation by flow cytometry of the intracellular ROS induced by 48 h of exposure of A549 cells to IC_50_ concentrations of compounds. (0.1% (v/v) DMSO was used as the solvent control, and 1.5 μM DOX was used as the positive control. ns not significant).

Reactive oxygen species (ROS), such as superoxide or hydrogen peroxide, are produced during physiological processes and are considered extremely reactive. These molecules could be secondary messengers involved in the regulation of cell adhesion or programmed cell death.^34^ In another perspective, this species could cause oxidative damage to fatty acids, DNA, RNA, or proteins, which could lead to several diseases.^35^Fig. 8 illustrates that the three MTX salts do not promote the formation of ROS (values of MTX salts equal to control values), concluding that their antiproliferative effect is not due to an increase in ROS.

## 4. Conclusions

Here, twelve MTX salts were prepared by sustainable acid–base neutralization reactions in high yields (87–98%) and then characterized by NMR and elemental analysis to elucidate their desired chemical structure. It is important to emphasize the large improvement in the water solubility profile of the novel MTX salts compared to initial MTX. Among the twelve MTX salts, three of them, [C_12_mim]_2_[MTX], [C_10_mim]_2_[MTX] and [C_10_-3-picoline]_2_[MTX], showed an antiproliferative effect in the KRAS-mutated A549 NSCLC cell line compared to the EGFR-mutated NSCLC cell line H1975. The order of cytotoxicity is [C_12_mim]_2_[MTX] (IC_50_ = 0.55 ± 0.25 μM) > [C_10_-3-picoline]_2_[MTX] (IC_50_ = 0.94 ± 0.03 μM) > [C_10_mim]_2_[MTX] (IC_50_ = 1.71 ± 0.23 μM). Moreover, the antiproliferative potential was higher than that of original MTX and sodium MTX salt. Interestingly, due to the structure–activity relationship, it is possible to conclude that longer alkyl chains (C_10_ and C_12_) conferred an antiproliferative effect but only when linked to an imidazole or picoline ring.

The three MTX salts also showed a higher SI towards A549 NSCLC cells (lower IC_50_) compared to that in normal fibroblasts. In general, [C_10_-3-picoline]_2_[MTX] (IC_50_ = 0.940 μM) is the most promising candidate compared to IC_50_ in fibroblast (10.62 μM) with a SI of 11.3. These three MTX salts are able to trigger intrinsic apoptosis (*via* mitochondria) without increasing ROS levels.

## Data availability

The data supporting this article have been included as part of the ESI.†

## Author contributions

DV and SC contribute with experimental, validation and writing initial draft. PVB, ARF and LCB were involved in the supervision, validation, funding and revising final article.

## Conflicts of interest

There are no conflicts to declare.

## Supplementary Material

MD-OLF-D4MD00960F-s001
