# Supplementary material for: Synthesis, characterization and biological activity of methotrexate-derived salts in lung cancer cells
Source: RSC Med Chem. 2025 Apr 16;16(8):3593–602. doi: 10.1039/d4md00960f (PMC12121626; doi:10.1039/d4md00960f)
Supplement: MD-016-D4MD00960F-s001 [file MD-016-D4MD00960F-s001.pdf]

Supplementary Information (SI) for RSC Medicinal Chemistry.
This journal is © The Royal Society of Chemistry 2025

Supporting information

Synthesis, characterization and biological assays of methotrexate-derived salts in lung cancer cells

Dário Silva[1,2,3], Sandra Cordeiro[2,3], Pedro V. Baptista[2,3] Alexandra R. Fernandes[2,3,*]and Luis C. Branco[1,*]

[1] LAQV-REQUIMTE, Nova School of Science and Technology, NOVA University Lisbon 2829-516 Caparica, Portugal
[2] Associate Laboratory i4HB - Institute for Health and Bioeconomy, NOVA School of Science and Technology, NOVA University Lisbon, 2819-516 Caparica, Portugal
[3] UCIBIO – Applied Molecular Biosciences Unit, Department of Life Sciences, NOVA School of Science and Technology, NOVA University Lisbon, 2819-516 Caparica, Portugal

*Co-last and corresponding authors

Table of Contents

NMR characterization of MTX salts		
$[Na]_2[MTX]$ ^1H NMR spectra	Fig.S1	Page 3
$[Na]_2[MTX]$ ^{13}C NMR spectra.	Fig.S2	Page 4
$[Choline]_2[MTX]$ ^1H NMR spectra	Fig.S3	Page 4
$[Choline]_2[MTX]$ ^{13}C NMR spectra	Fig.S4	Page 5
$[N_{12,1,1,2OH}]_2[MTX]$ ^1H NMR spectra	Fig.S5	Page 5
$[N_{12,1,1,2OH}]_2[MTX]$ ^{13}C NMR spectra	Fig.S6	Page 6
$[N_{10,1,1,2OH}]_2[MTX]$ ^1H NMR spectra	Fig.S7	Page 6
$[N_{10,1,1,2OH}]_2[MTX]$ ^{13}C NMR spectra	Fig.S8	Page 7
$[C_2mim]_2[MTX]$ ^1H NMR spectra	Fig.S9	Page 7
$[C_2mim]_2[MTX]$ ^{13}C NMR spectra	Fig.S10	Page 8
$[C_2OHmim]_2[MTX]$ ^1H NMR spectra	Fig.S11	Page 8
$[C_2OHmim]_2[MTX]$ ^{13}C NMR spectra	Fig.S12	Page 9
$[C_6mim]_2[MTX]$ ^1H NMR spectra	Fig.S13	Page 9
$[C_6mim]_2[MTX]$ ^{13}C NMR spectra	Fig.S14	Page 10
$[C_8mim]_2[MTX]$ ^1H NMR spectra	Fig.S15	Page 10
$[C_8mim]_2[MTX]$ ^{13}C NMR spectra	Fig.S16	Page 11
$[C_{10}mim]_2[MTX]$ ^1H NMR spectra	Fig.S17	Page 11
$[C_{10}mim]_2[MTX]$ ^{13}C NMR spectra	Fig.S18	Page 12
$[C_{12}mim]_2[MTX]$ ^1H NMR spectra	Fig.S19	Page 12
$[C_{12}mim]_2[MTX]$ ^{13}C NMR spectra	Fig.S20	Page 13
$[C_2OH$ 3-picoline$]_2[MTX]$ ^1H NMR spectra	Fig.S21	Page 13
$[C_2OH$ 3-picoline$]_2[MTX]$ ^{13}C NMR spectra	Fig.S22	Page 14
$[C_{10}$ 3-picoline$]_2[MTX]$ ^1H NMR spectra	Fig.S23	Page 14
$[C_{10}$ 3-picoline$]_2[MTX]$ ^{13}C NMR spectra	Fig.S24	Page 15
$[MIMC_{12}MIM]_2[MTX]$ ^1H NMR spectra	Fig.S25	Page 15
$[MIMC_{12}MIM]_2[MTX]$ ^{13}C NMR spectra	Fig.S26	Page 16
ATF-FTIR spectra of $[Choline]_2[MTX]$	Fig.S27	Page 16
ATF-FTIR spectra of $[N_{12,1,1,2OH}]_2[MTX]$	Fig.S28	Page 17
ATF-FTIR spectra of $[N_{10,1,1,2OH}]_2[MTX]$	Fig.S29	Page 17

ATF-FTIR spectra of $[C_2mim]_2[MTX]$	Fig.S30	Page 18
ATF-FTIR spectra of $[C_2OHmim]_2[MTX]$	Fig.S31	Page 18
ATF-FTIR spectra of $[C_6mim]_2[MTX]$	Fig.S32	Page 19
ATF-FTIR spectra of $[C_8mim]_2[MTX]$	Fig.S33	Page 19
ATF-FTIR spectra of $[C_{10}mim]_2[MTX]$	Fig.S34	Page 20
ATF-FTIR spectra of $[C_{12}mim]_2[MTX]$	Fig.S35	Page 20
ATF-FTIR spectra of $[C_2OH$ 3-picoline$]_2[MTX]$	Fig.S36	Page 21
ATF-FTIR spectra of $[C_{10}$ 3-picoline$]_2[MTX]$	Fig.S37	Page 21
ATF-FTIR spectra of $[MIMC_{12}MIM]_2[MTX]$	Fig.S38	Page 22
UV/Vis spectrum of A. MTX and B. $[Na]_2[MTX]$ in DMEM after 0, 24 and 48h at 37 ^{0}C	Fig.S39	Page 22
**Biological Studies**		
Cell viability of A549 cell line after exposure to different concentrations of the $[Na]_2[MTX]$ for 48 hours. 0.1% (v/v) DMSO was used as the vehicle control. (**** $p < 0.0001$).	Fig.S40	Page 23
Cell viability of A549 cell line after exposure to different concentrations of the $[N_{12,1,1,2OH}]_2[MTX]$ for 48 hours. 0.1% (v/v) DMSO was used as the vehicle control. (**** $p < 0.0001$).	Fig.S41	Page 23
Cell viability of A549 cell line after exposure to different concentrations of the $[N_{10,1,1,2OH}]_2[MTX]$ for 48 hours. 0.1% (v/v) DMSO was used as the vehicle control. (*** $p < 0.001$; **** $p < 0.0001$).	Fig.S42	Page 24
Cell viability of A549 cell line after exposure to different concentrations of the $[MIMC_{12}MIM]_2[MTX]$ for 48 hours. 0.1% (v/v) DMSO was used as the vehicle control. (** $p < 0.01$; **** $p < 0.0001$).	Fig.S43	Page 24
Cell viability of A549 cell line after exposure to different concentrations of the $[C_{12}mim][Br]$ for 48 hours. 0.1% (v/v) DMSO was used as the vehicle control. (* $p < 0.05$; **** $p < 0.0001$).	Fig.S44	Page 25
Cell viability of A549 cell line after exposure to different concentrations of the $[C_{10}mim][Br]$ for 48 hours. 0.1% (v/v) DMSO was used as the vehicle control. (*** $p < 0.001$; **** $p < 0.0001$).	Fig.S45	Page 25
Cell viability of A549 cell line after exposure to different concentrations of the $[C_{10}$ 3-picoline$][Br]$ for 48 hours. 0.1% (v/v) DMSO was used as the vehicle control. (**** $p < 0.0001$).	Fig.S46	Page 26
Cell viability of H1975 cell line after exposure to different concentrations of the $[C_{12}mim]_2[MTX]$ for 48 hours. 0.1% (v/v) DMSO was used as the vehicle control. (**** $p < 0.0001$).	Fig.S47	Page 26
Cell viability of H1975 cell line after exposure to different concentrations of the $[C_{10}mim]_2[MTX]$ for 48 hours. 0.1% (v/v) DMSO was used as the vehicle control. (**** $p < 0.0001$).	Fig.S48	Page 27
Cell viability of H1975 cell line after exposure to different concentrations of the $[C_{10}$ 3-picoline$]_2[MTX]$ for 48 hours. 0.1% (v/v) DMSO was used as the vehicle control. (**** $p < 0.0001$).	Fig.S49	Page 27
Cell viability of H1975 cell line after exposure to different concentrations of the $[C_{12}mim][Br]$ for 48 hours. 0.1% (v/v) DMSO was used as the vehicle control. (* $p < 0.05$; ** $p < 0.01$; **** $p < 0.0001$).	Fig.S50	Page 28
Cell viability of H1975 cell line after exposure to different concentrations of the $[C_{10}mim][Br]$ for 48 hours. 0.1% (v/v) DMSO was used as the vehicle control. (**** $p < 0.0001$)	Fig.S51	Page 28
Cell viability of H1975 cell line after exposure to different concentrations of the $[C_{10}$ 3-picoline$][Br]$ for 48 hours. 0.1% (v/v) DMSO was used as the vehicle control. (*** $p < 0.001$; **** $p < 0.0001$).	Fig.S52	Page 29
Cell viability of Fibroblasts after exposure to different concentrations of the $[C_{12}mim]_2[MTX]$ for 48 hours. 0.1% (v/v) DMSO was used as the vehicle control. (* $p < 0.05$; **** $p < 0.0001$).	Fig.S53	Page 29
Cell viability of Fibroblasts after exposure to different concentrations of the $[C_{10}mim]_2[MTX]$ for 48 hours. 0.1% (v/v) DMSO was used as the	Fig.S54	Page 30

vehicle control. (* $p < 0.05$; ** $p < 0.01$; *** $p < 0.001$; **** $p < 0.0001$).		
Cell viability of Fibroblasts after exposure to different concentrations of the [C_{10} 3-picoline]$_2$[MTX] for 48 hours. 0.1% (v/v) DMSO was used as the vehicle control. (**** $p < 0.0001$).	Fig.S55	Page 30
Cell viability of Fibroblasts after exposure to different concentrations of the [C_{12}mim][Br] for 48 hours. 0.1% (v/v) DMSO was used as the vehicle control. (** $p < 0.01$; **** $p < 0.0001$).	Fig.S56	Page 31
2D FACS plots for the apoptosis assay. R16 correspond to live cells, R14 cells in initial apoptosis, R15 cells in late apoptosis and R17 cells in necrosis. DS2 correspond to [C_{12}mim]$_2$[MTX], DS4 correspond to [C_{10}mim]$_2$[MTX] and DS5 correspond to [C_{10} 3-picoline]$_2$ [MTX].	Fig.S57	Page 32

NMR characterization of MTX salts

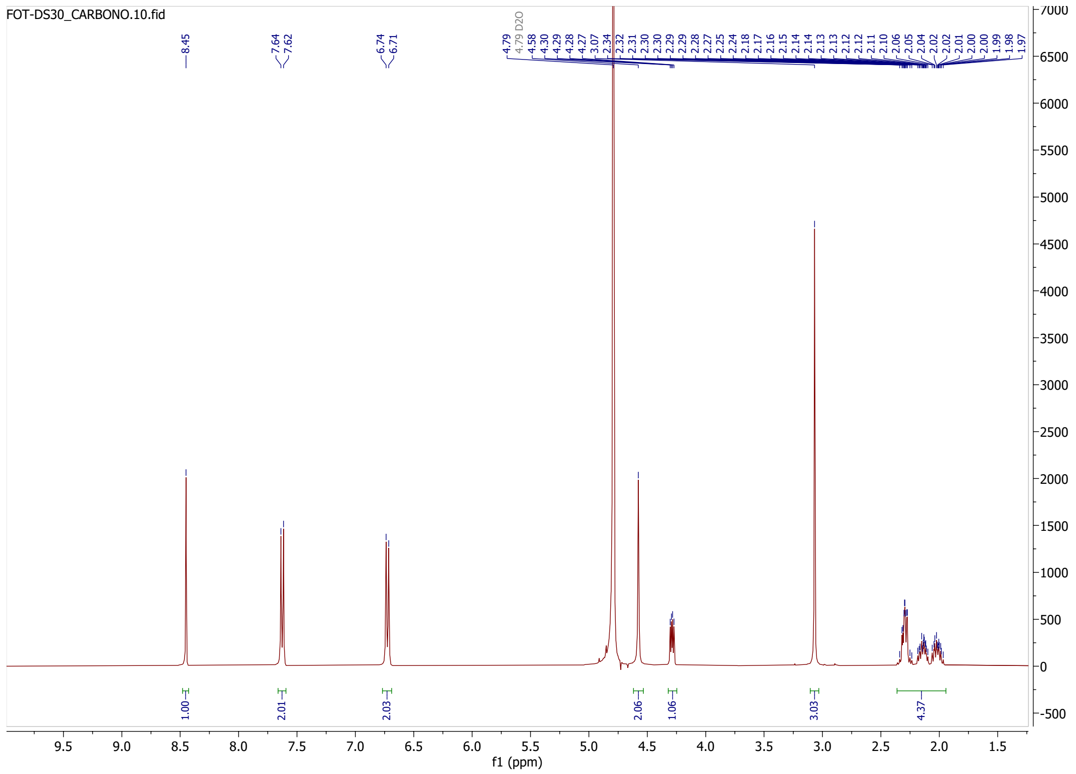

Fig.S1 – [Na]$_2$[MTX] ^{1}H NMR spectra.

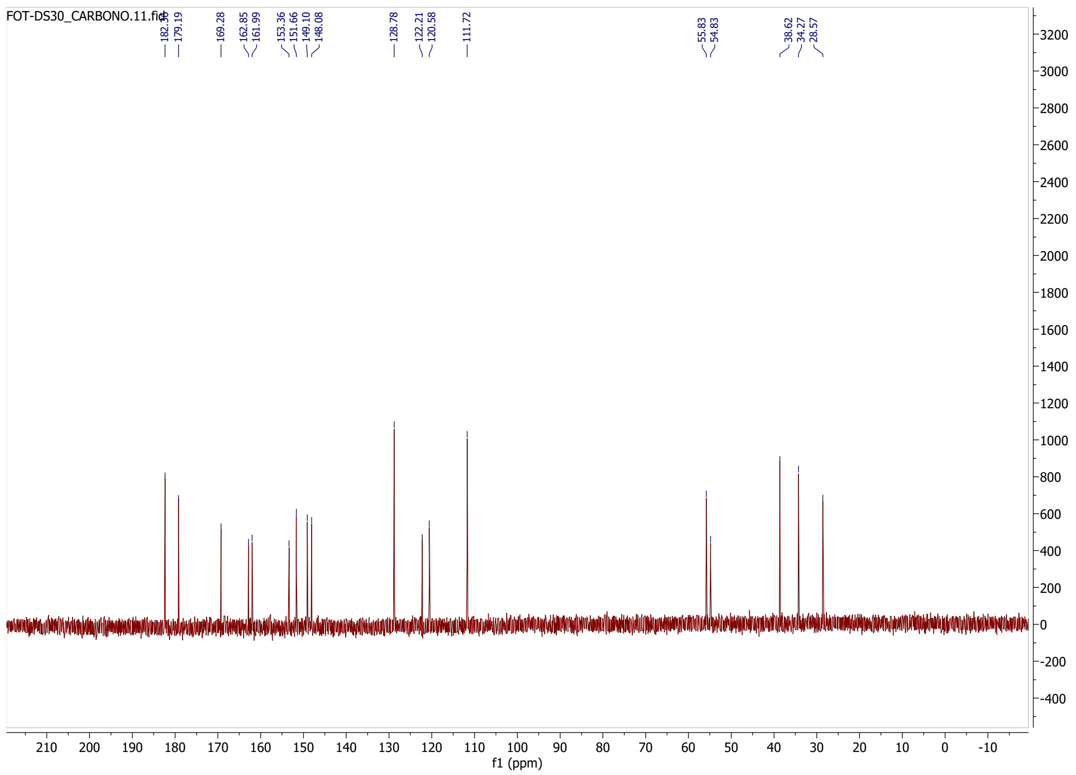

Fig.S2 – $[Na]_2[MTX]$ ^{13}C NMR spectra.

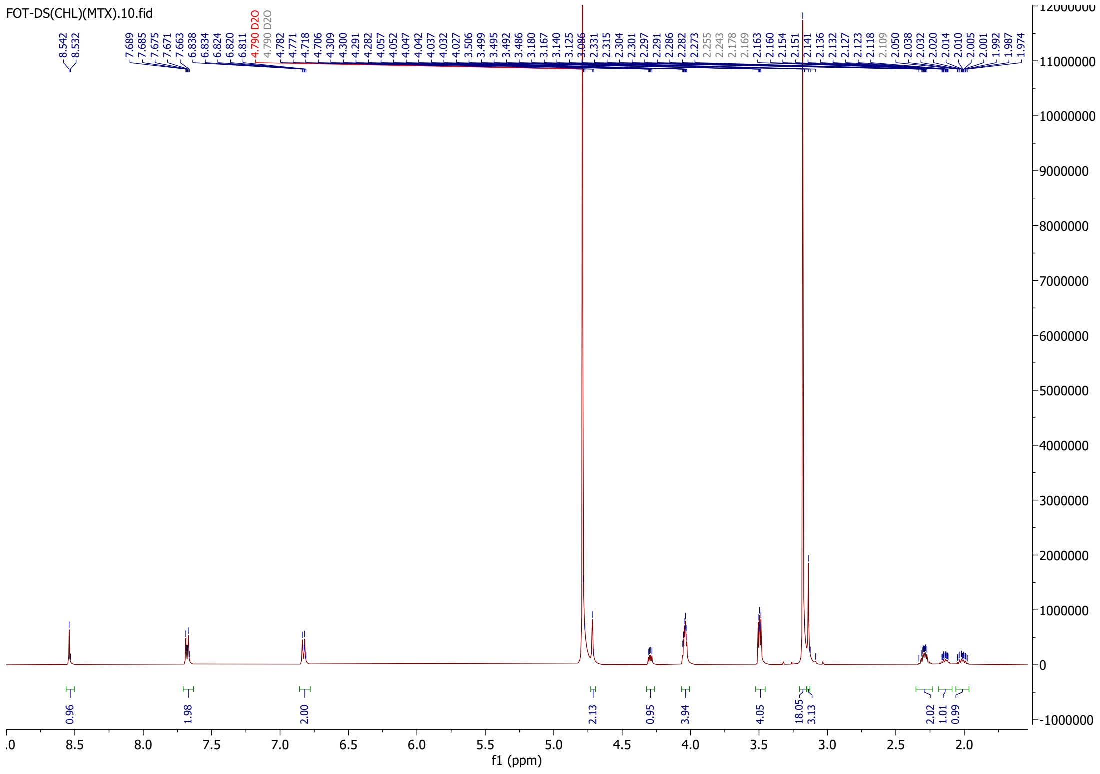

Fig.S3 – $[Choline]_2[MTX]$ ^{1}H NMR spectra.

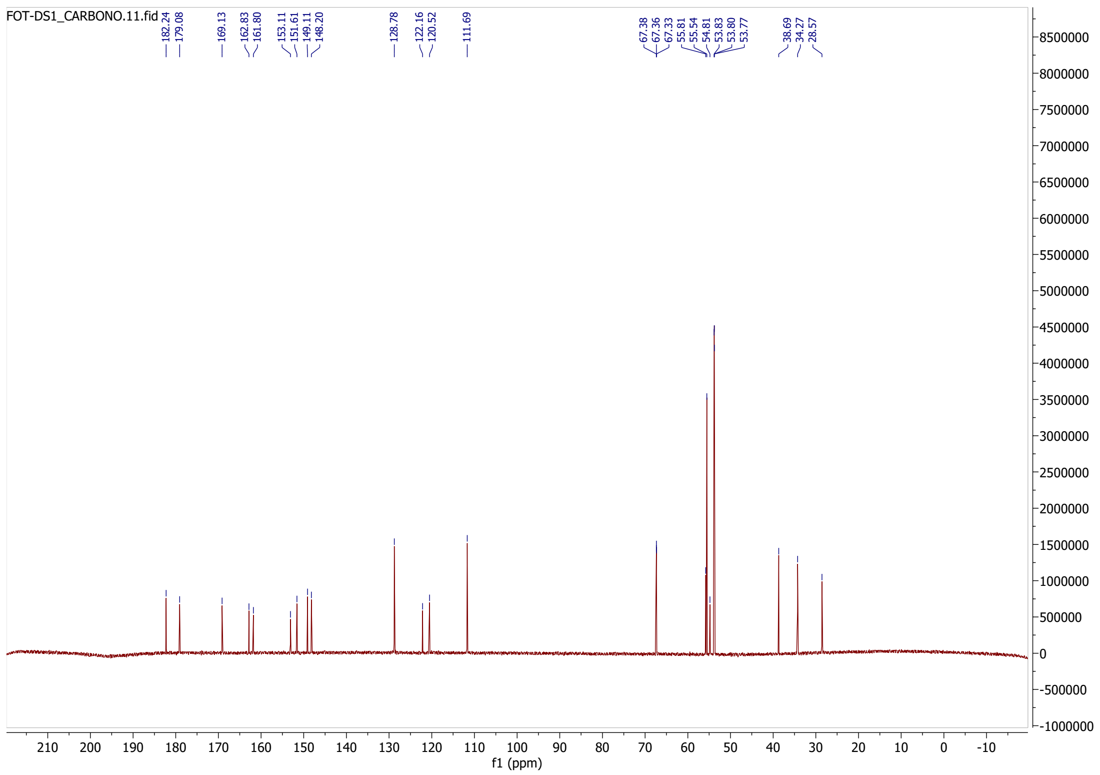

Fig.S4 – [Choline]$_2$[MTX] ^{13}C NMR spectra.

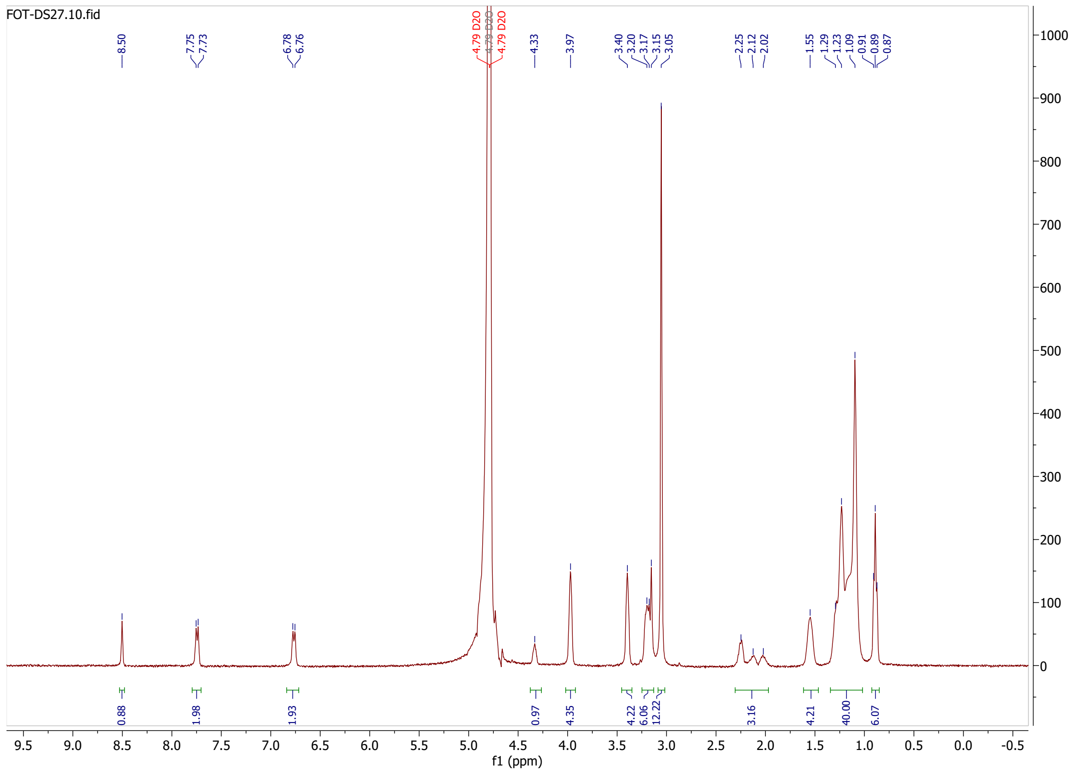

Fig.S5 – [$N_{12,1,1,2OH}$]$_2$[MTX] ^{1}H NMR spectra.

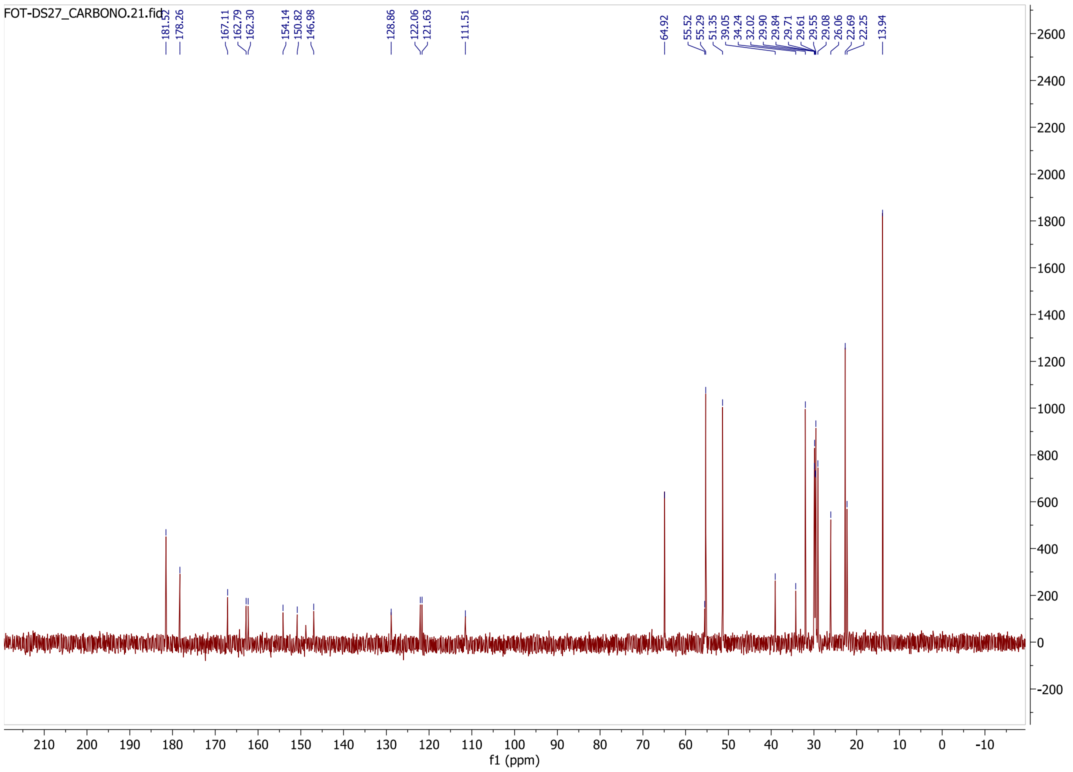

Fig.S6 – $[N_{12,1,1,2OH}]_2$[MTX] ^{13}C NMR spectra.

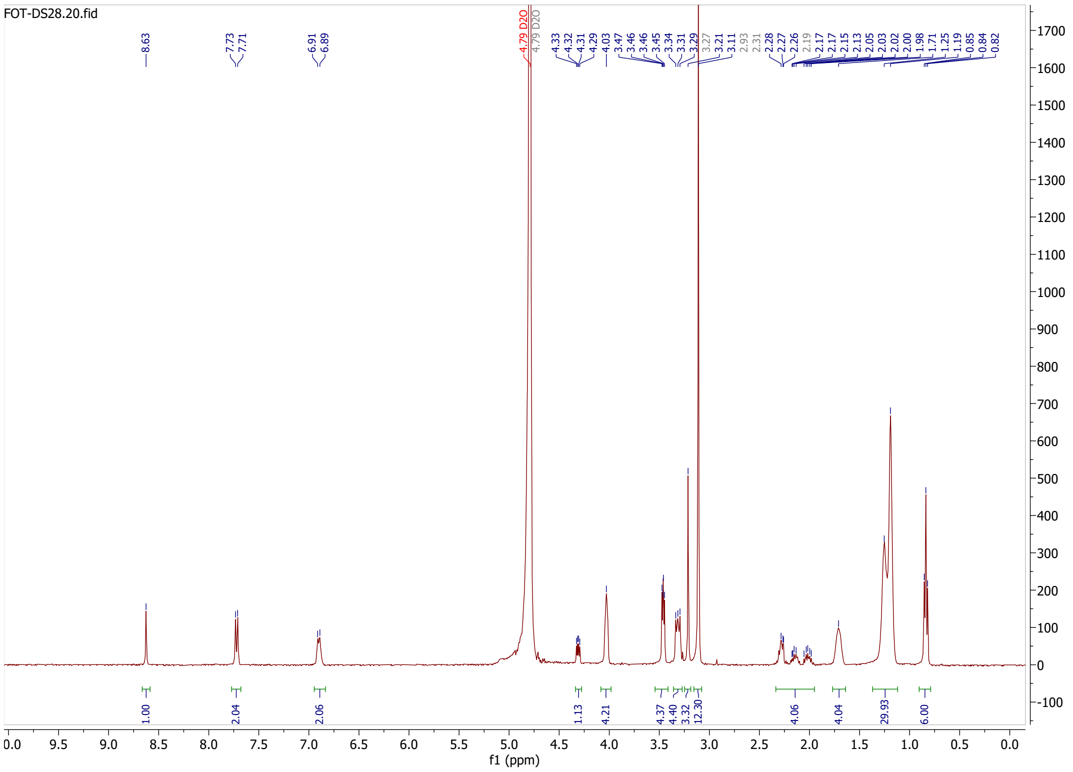

Fig.S7 – $[N_{10,1,1,2OH}]_2$[MTX] ^{1}H NMR spectra.

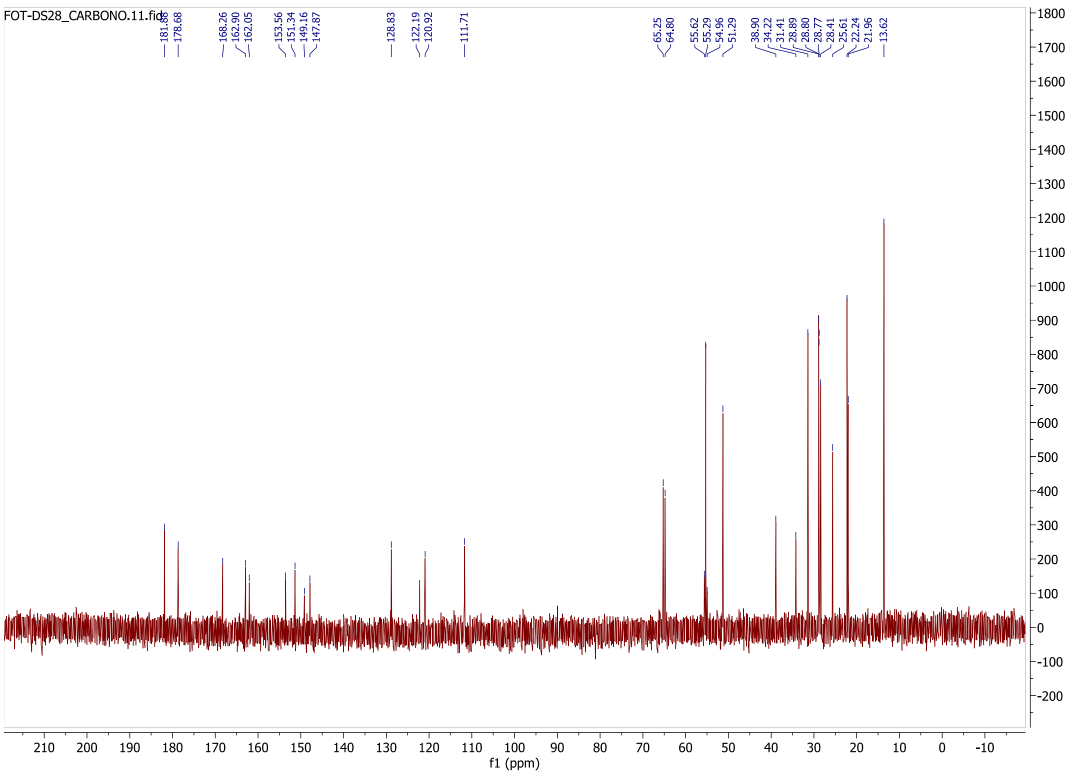

Fig.S8 – $[N_{10,1,1,2OH}]_2$[MTX] ^{13}C NMR spectra.

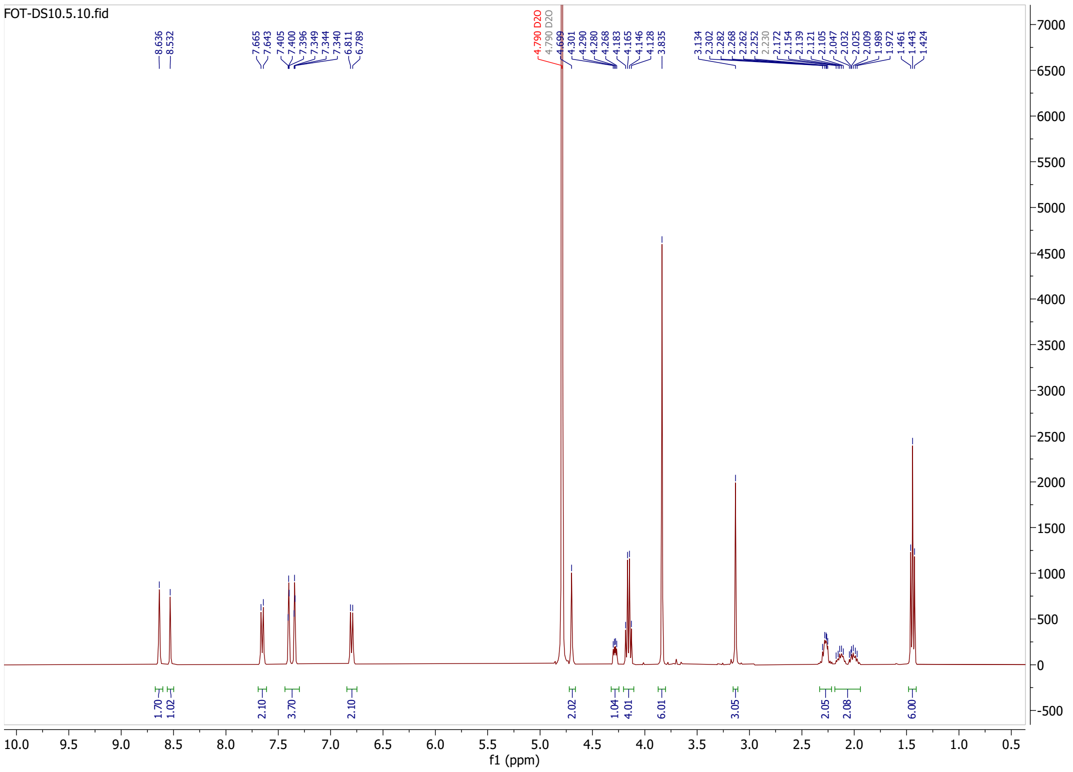

Fig.S9 – $[C_2mim]_2$[MTX] ^{1}H NMR spectra.

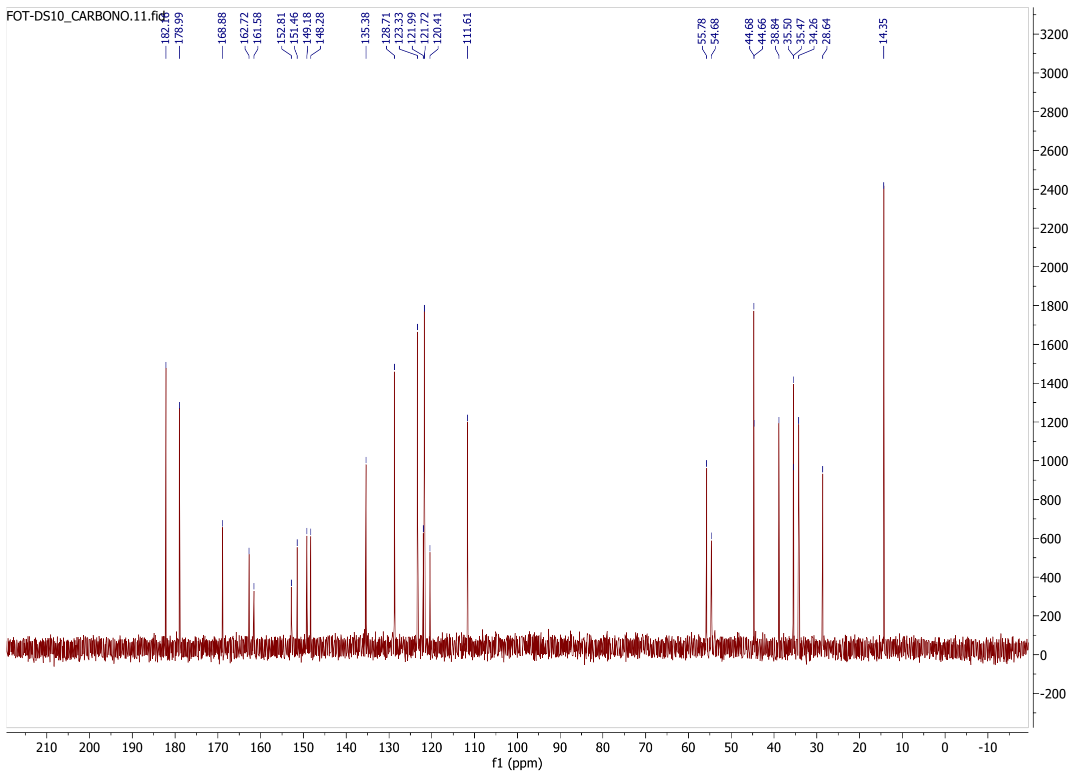

Fig.S10 – $[C_2mim]_2[MTX]$ ^{13}C NMR spectra.

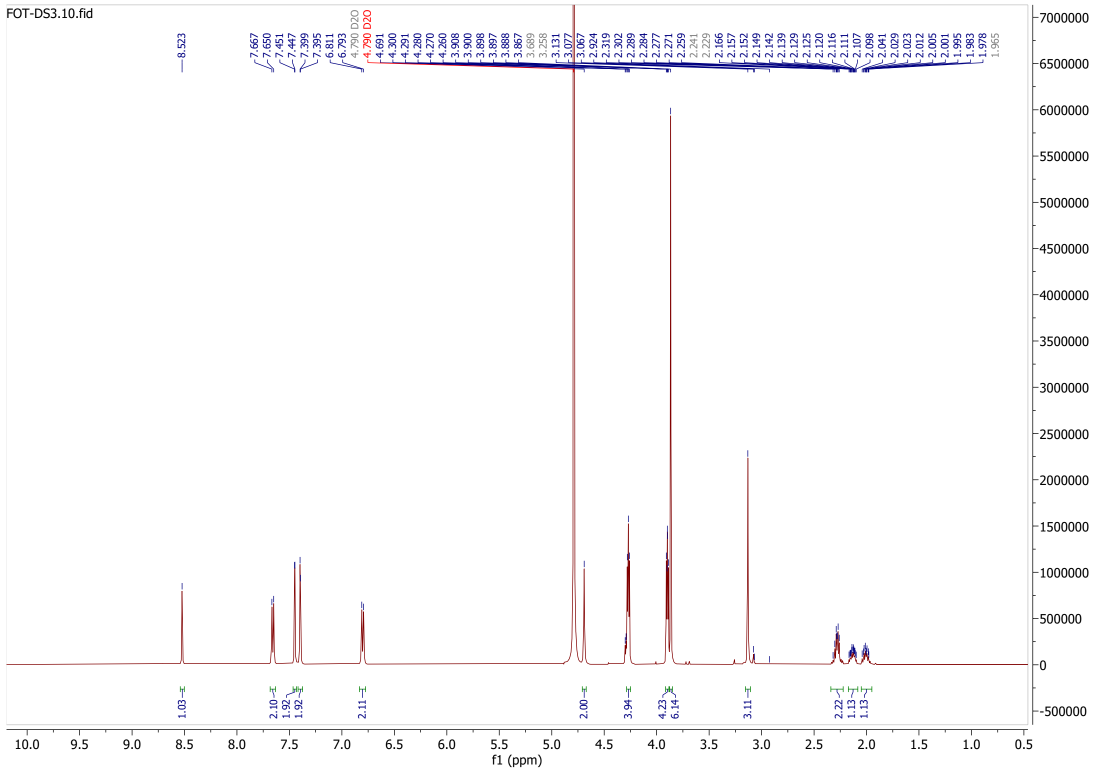

Fig.S11 – $[C_2OHmim]_2[MTX]$ ^{1}H NMR spectra.

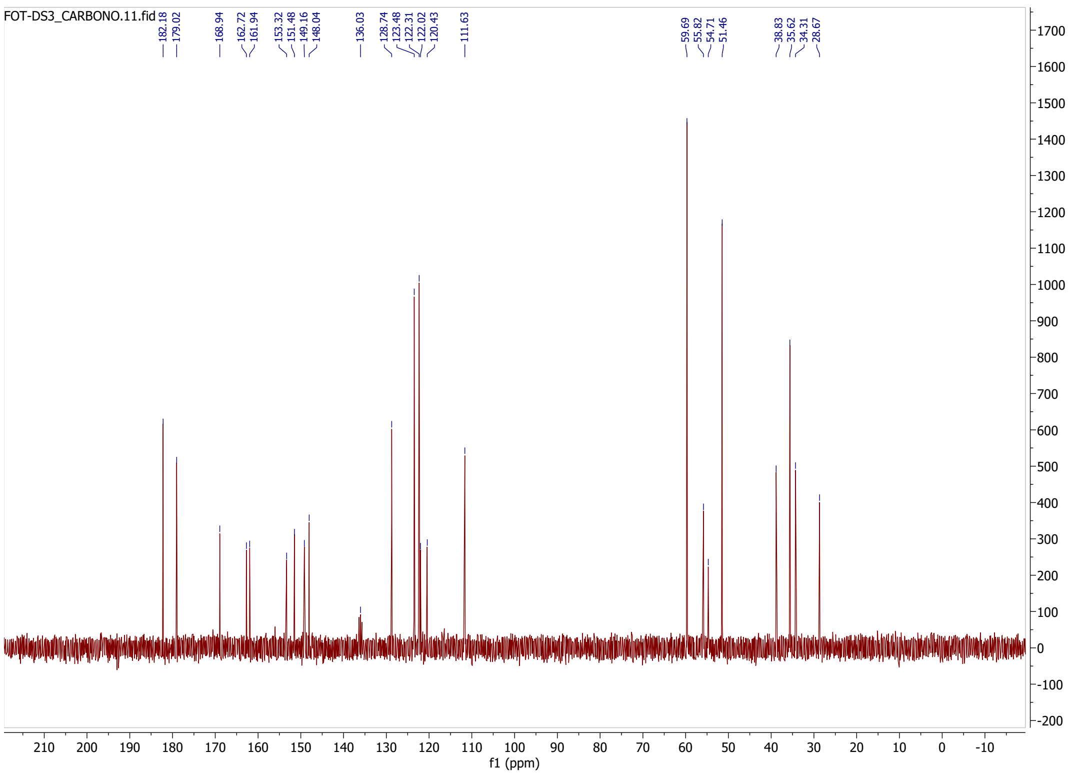

Fig.S12 – $[C_2OHmim]_2[MTX]$ ^{13}C NMR spectra.

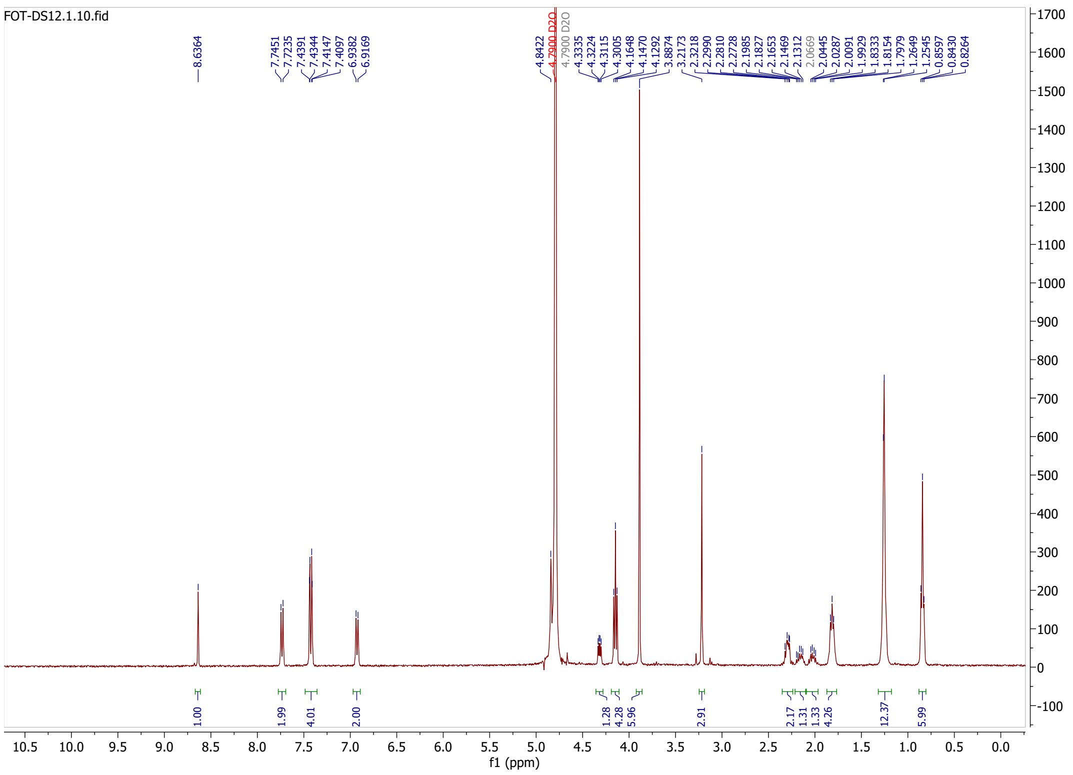

Fig.S13 – $[C_6mim]_2[MTX]$ ^{1}H NMR spectra.

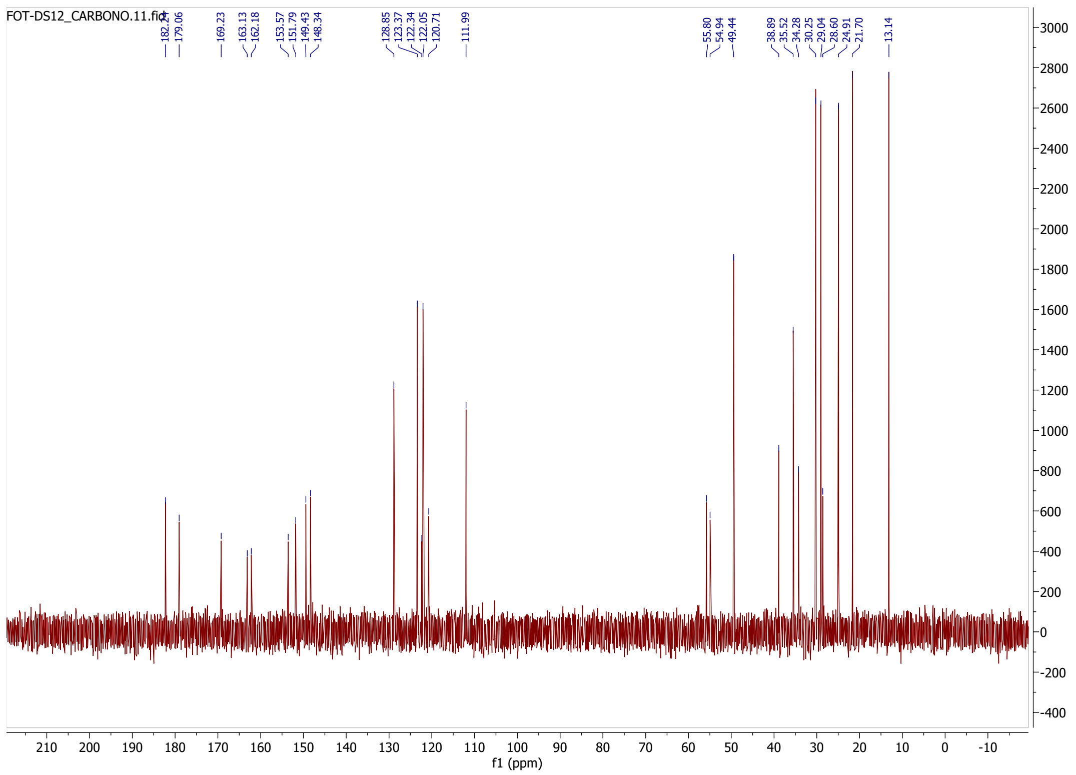

Fig.S14 – $[C_6mim]_2[MTX]$ ^{13}C NMR spectra.

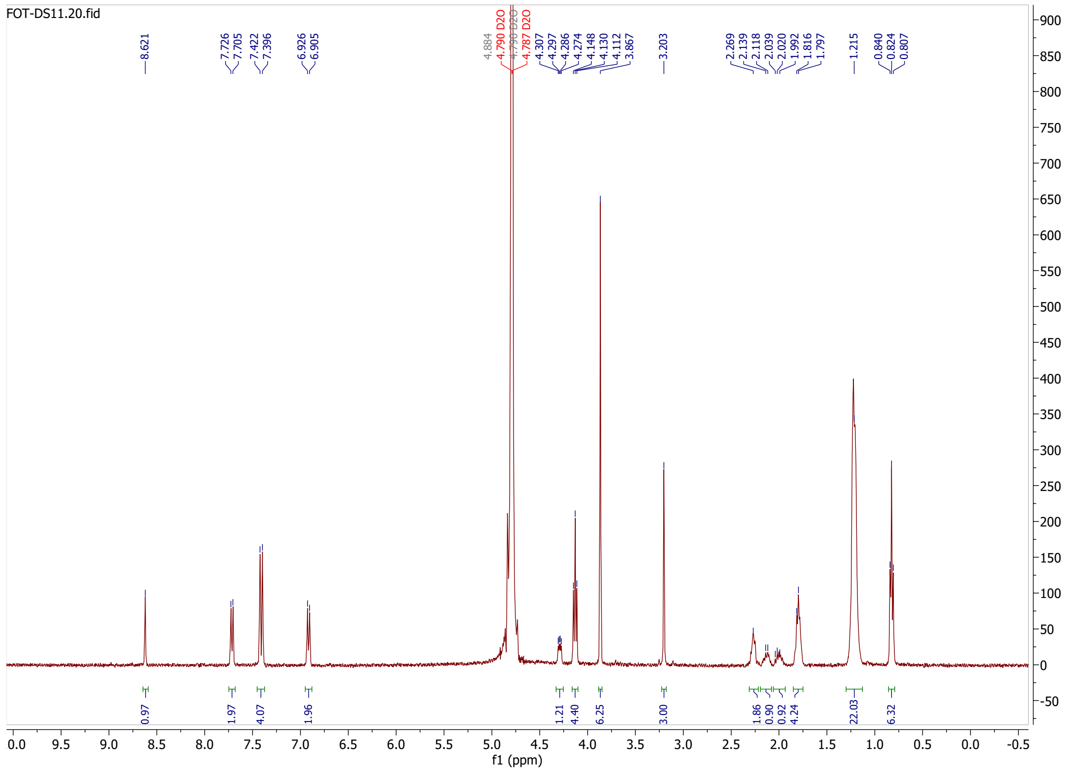

Fig.S15 – $[C_8mim]_2[MTX]$ ^{1}H NMR spectra.

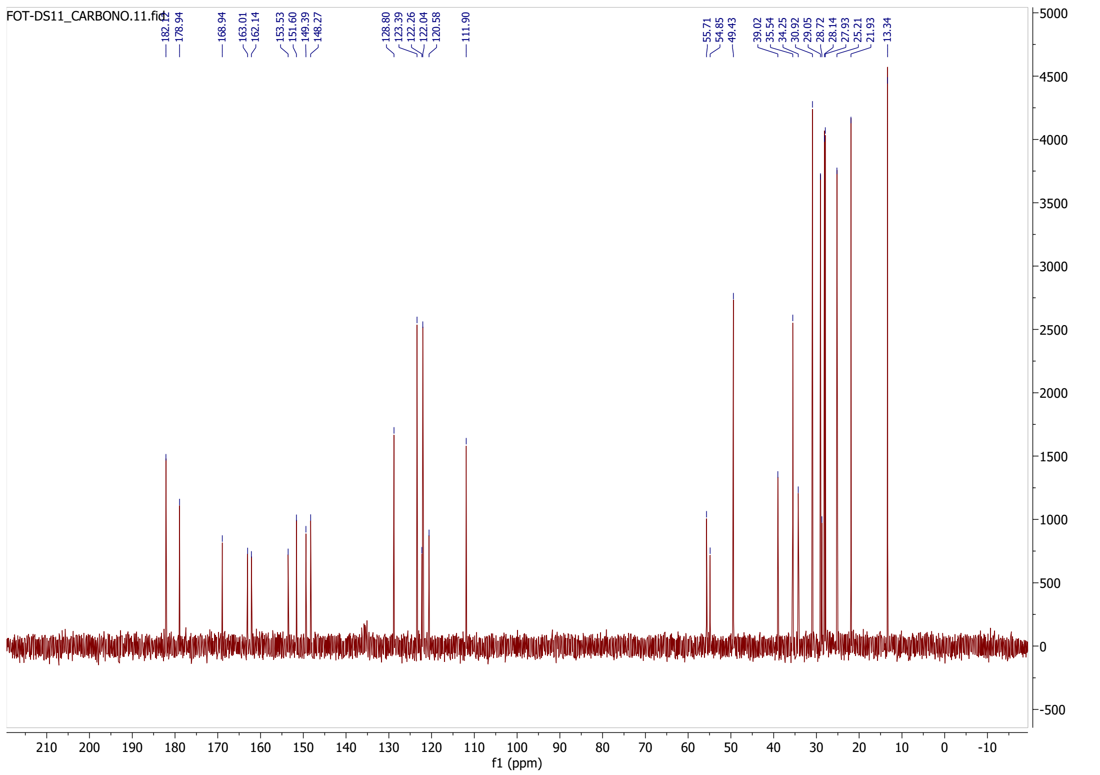

Fig.S16 – [C_8mim]$_2$[MTX] ^{13}C NMR spectra.

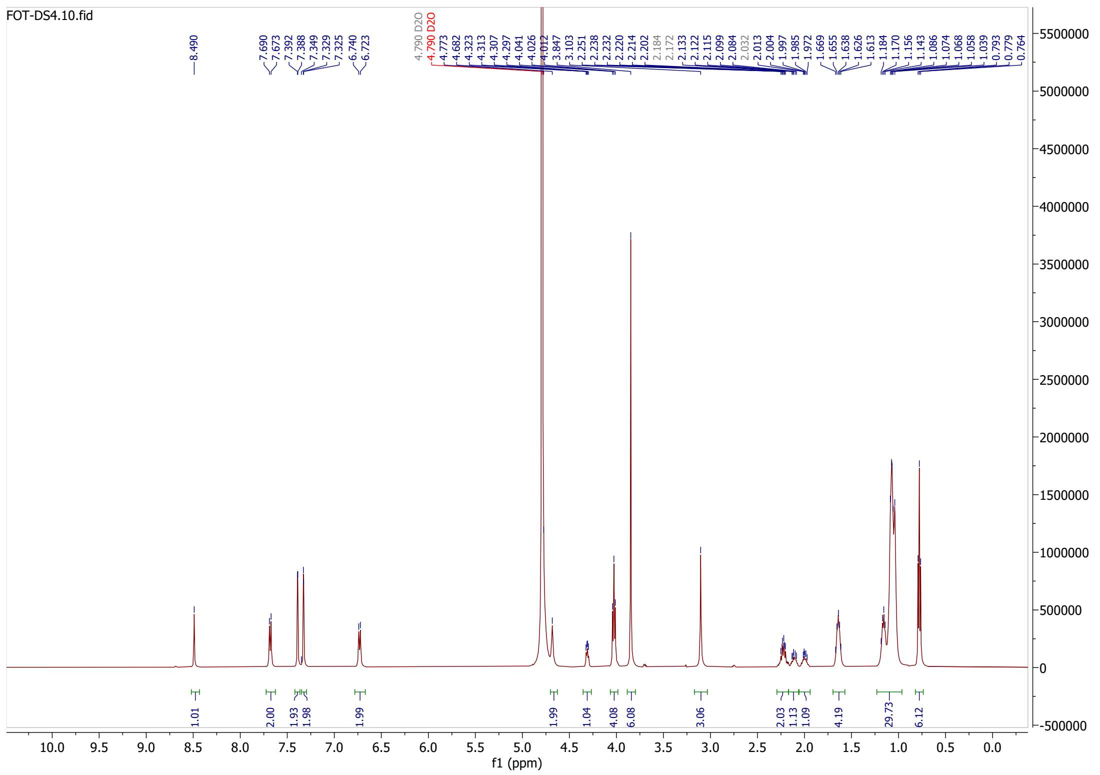

Fig.S17 – [C_{10}mim]$_2$[MTX] ^{1}H NMR spectra.

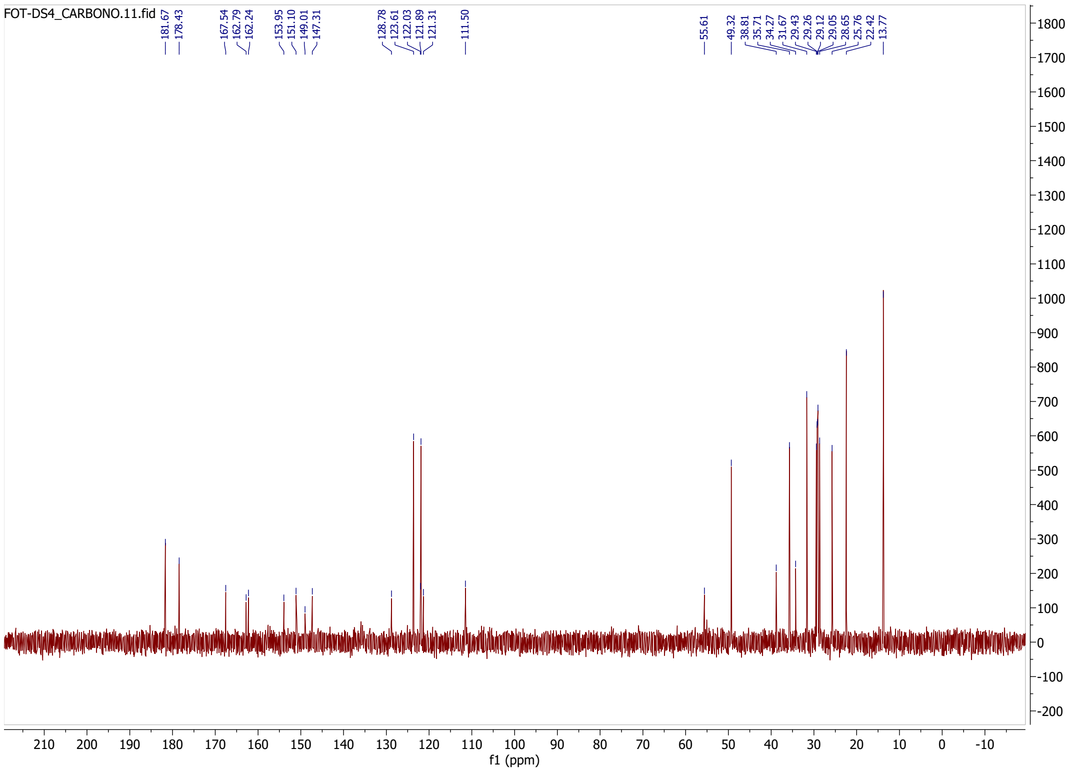

Fig.S18 – $[C_{10}mim]_2[MTX]$ ^{13}C NMR spectra.

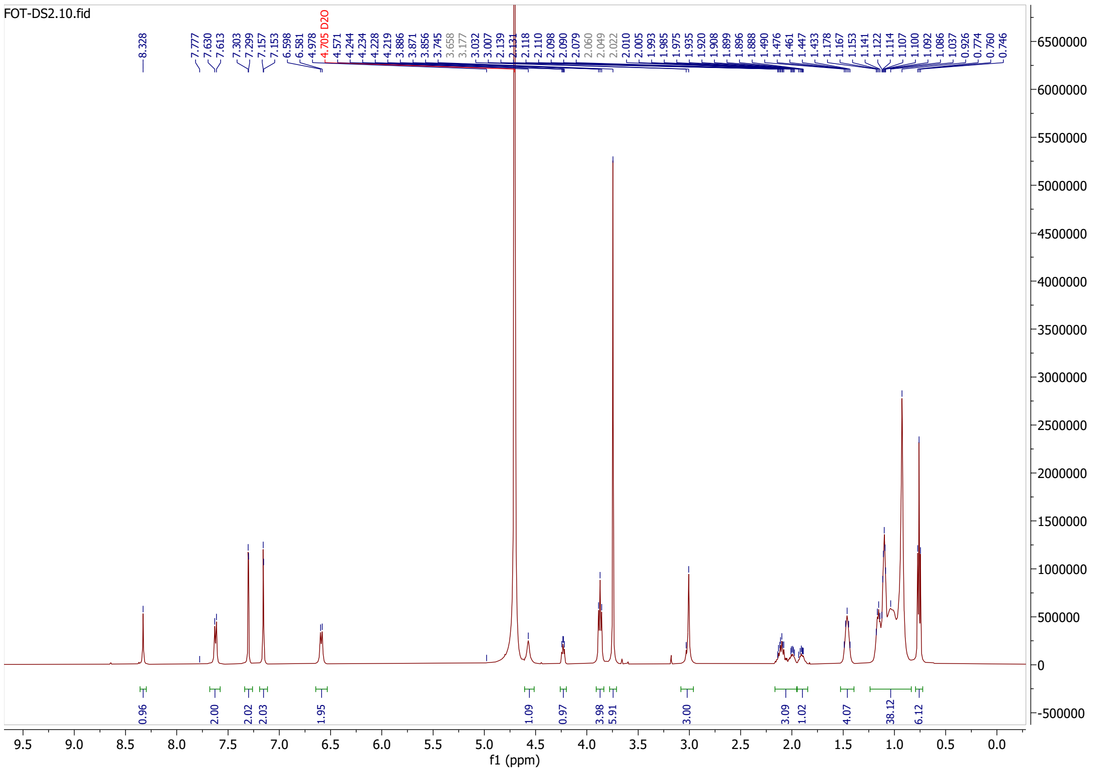

Fig.S19 – $[C_{12}mim]_2[MTX]$ ^{1}H NMR spectra.

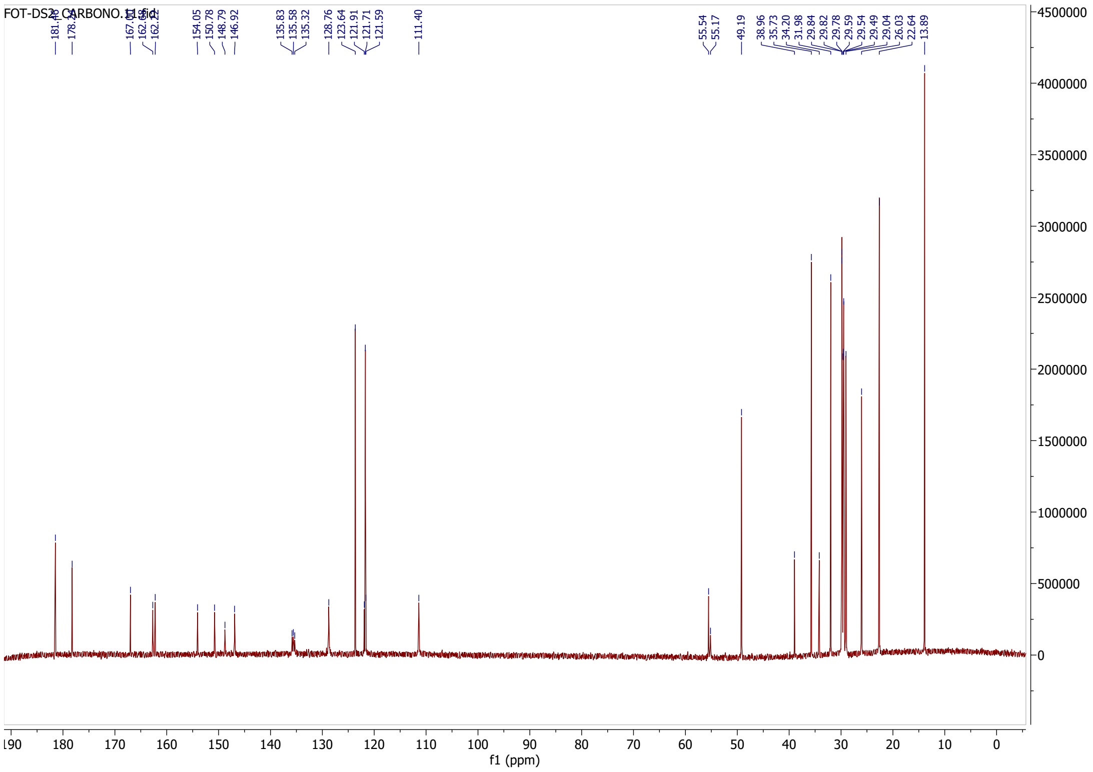

Fig.S20 – $[C_{12}mim]_2[MTX]$ ^{13}C NMR spectra.

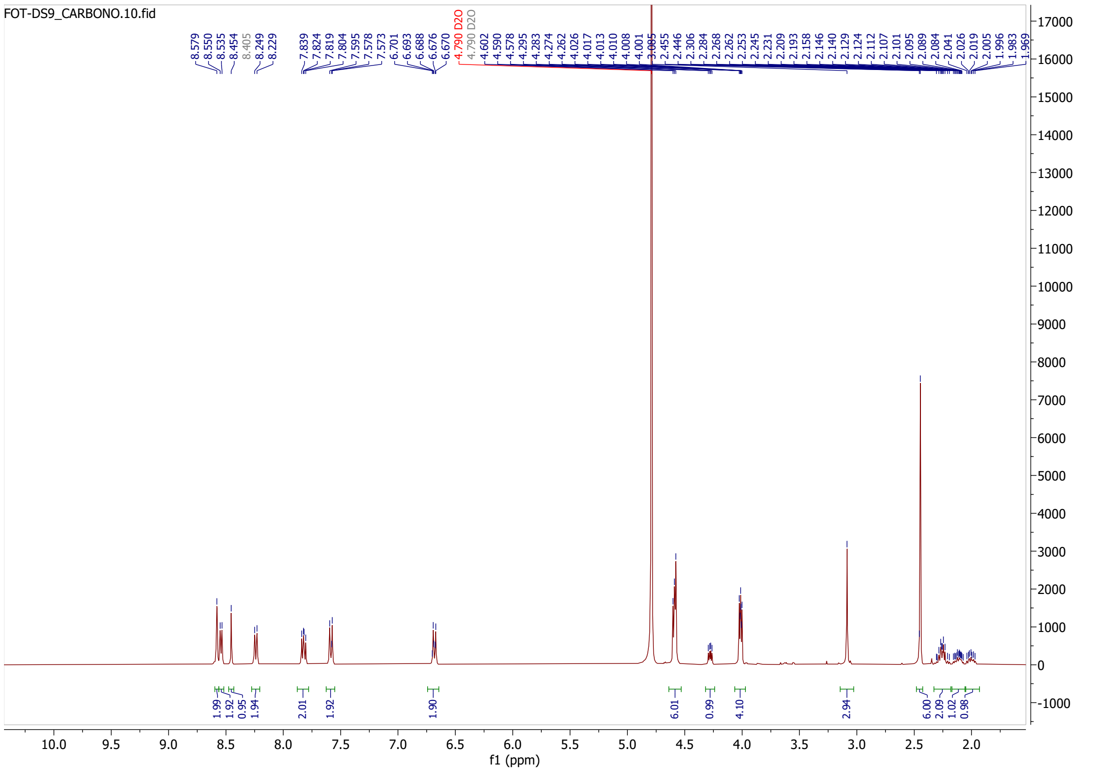

Fig.S21 – $[C_2OH$ 3-picoline$]_2[MTX]$ ^1H NMR spectra.

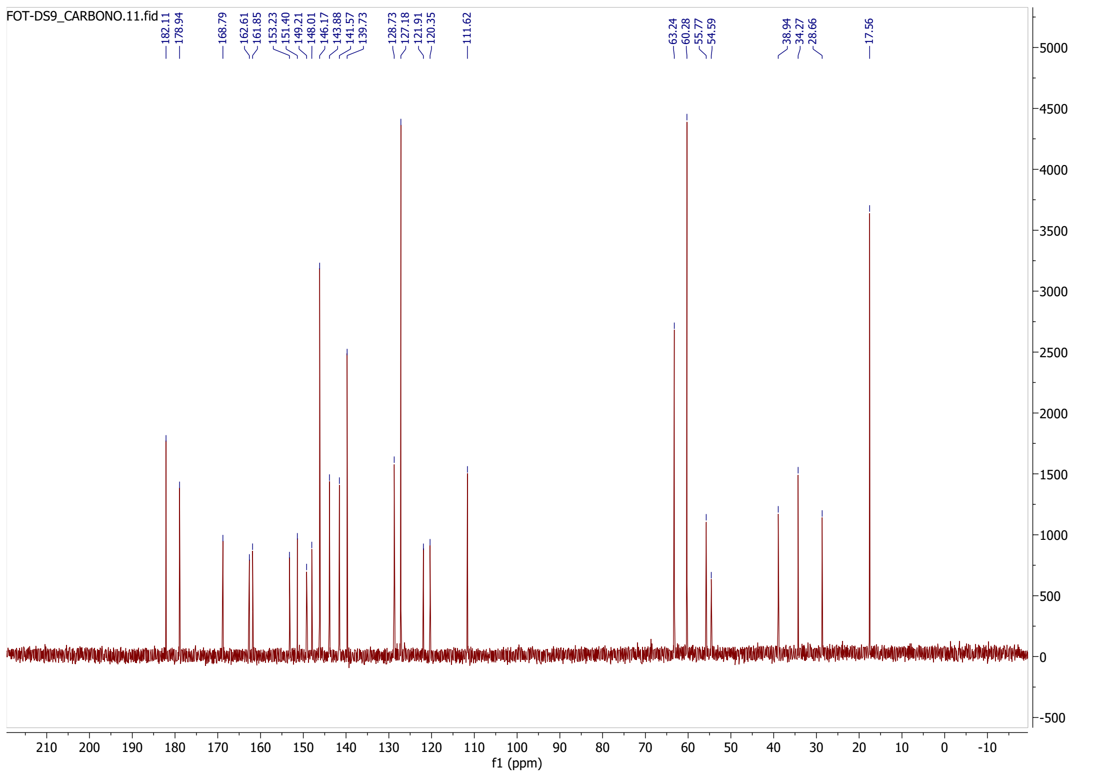

Fig.S22 – [C_2OH 3-picoline]$_2$[MTX] ^{13}C NMR spectra.

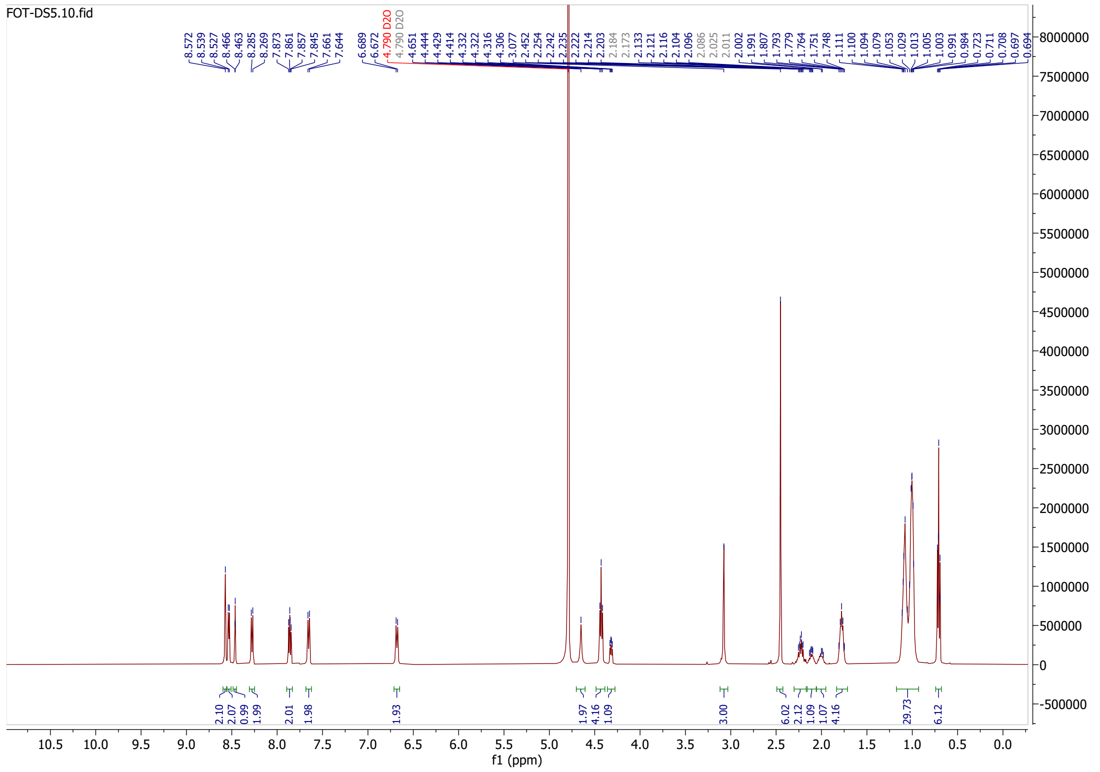

Fig.S23 – [C_{10} 3-picoline]$_2$[MTX] ^{1}H NMR spectra.

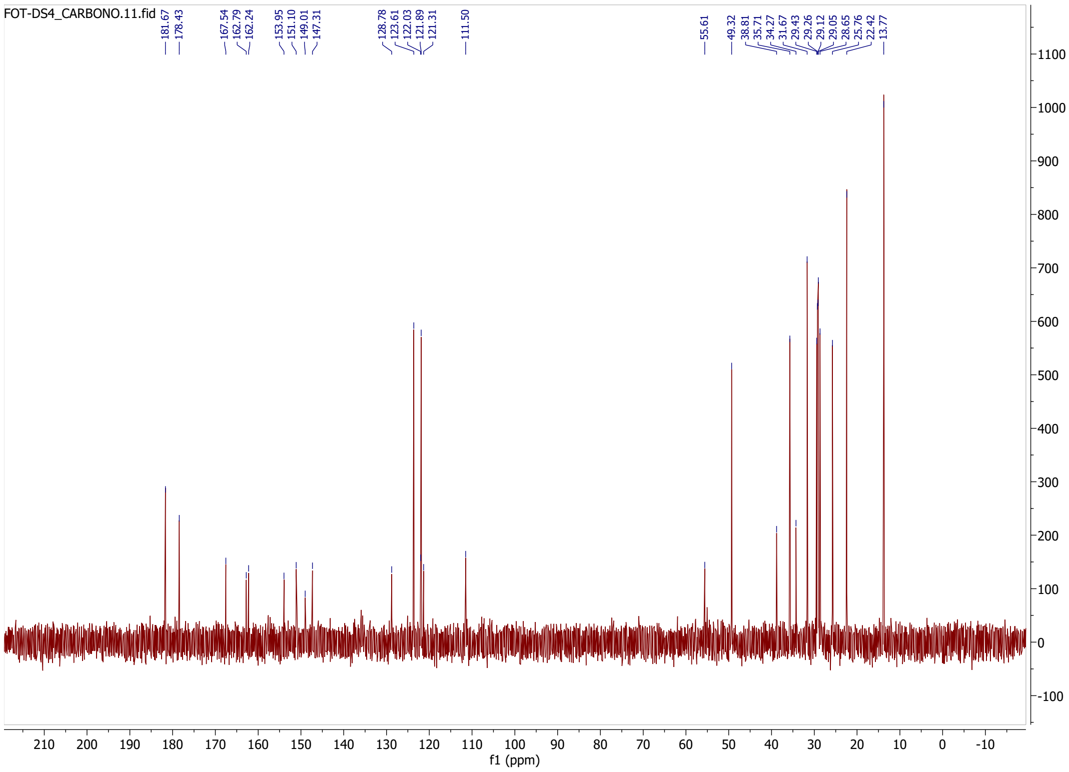

Fig.S24 – [C_{10} 3-picoline]$_2$[MTX] ^{13}C NMR spectra

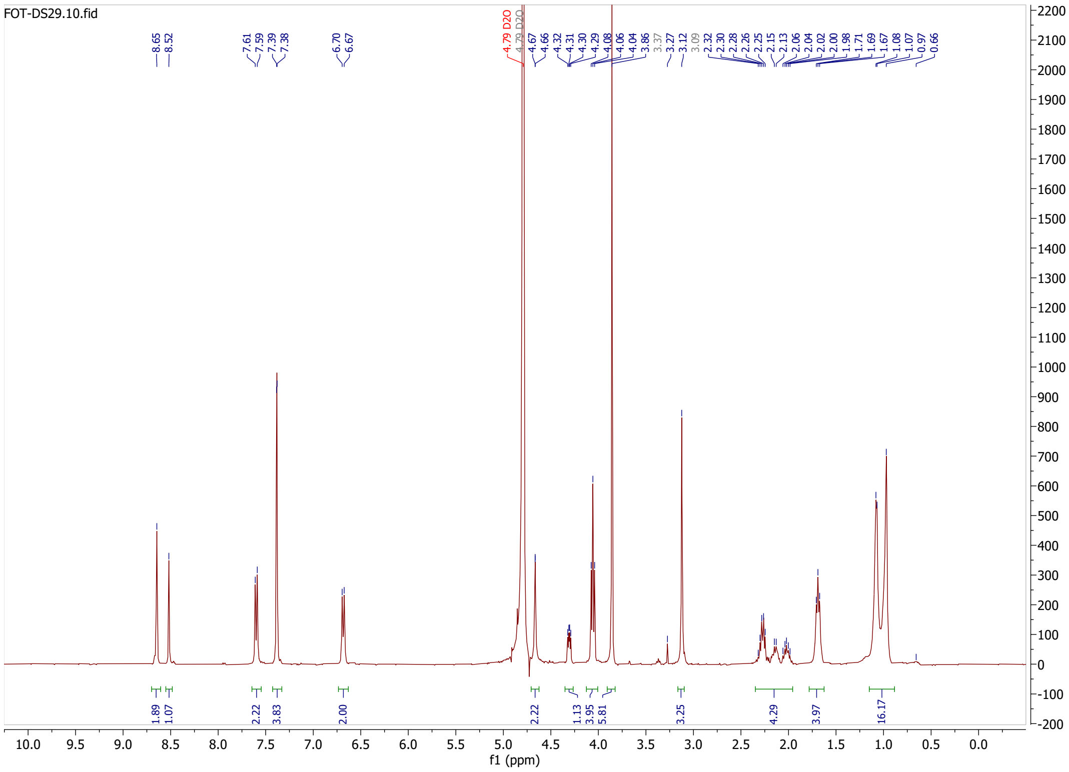

Fig.S25 – [$MIMC_{12}MIM$]$_2$[MTX] ^1H NMR spectra

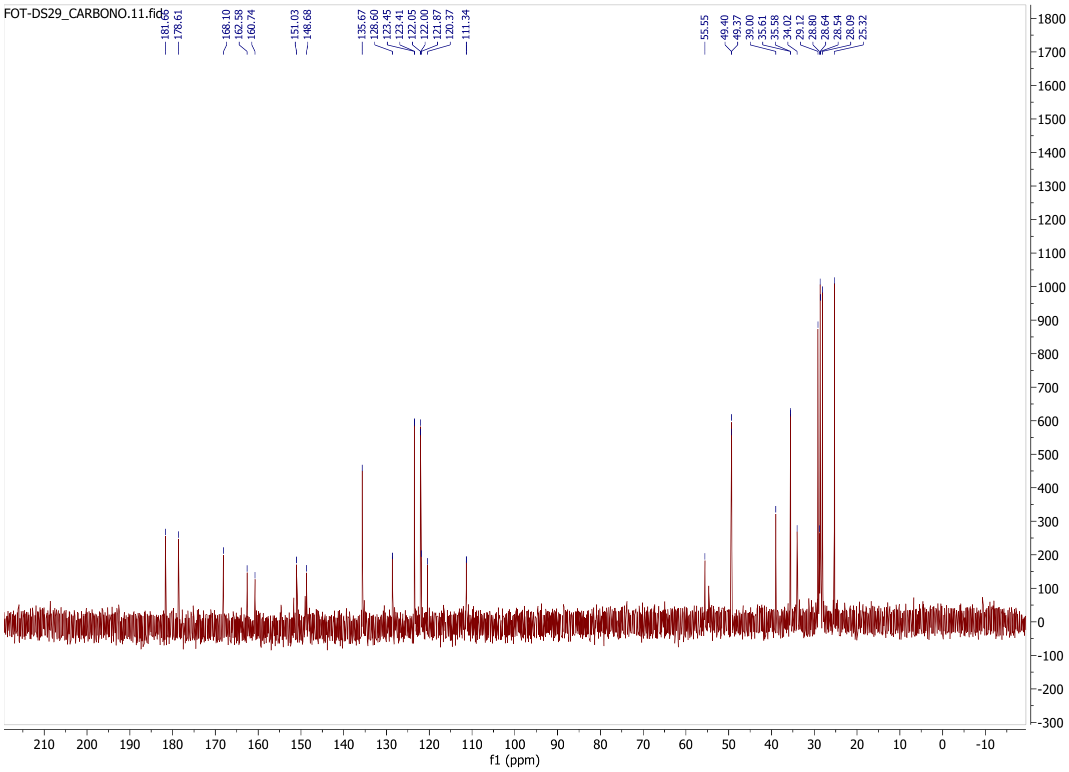

Fig.S26 – $[MIMC_{12}MIM]_2[MTX]$ ^{13}C NMR spectra

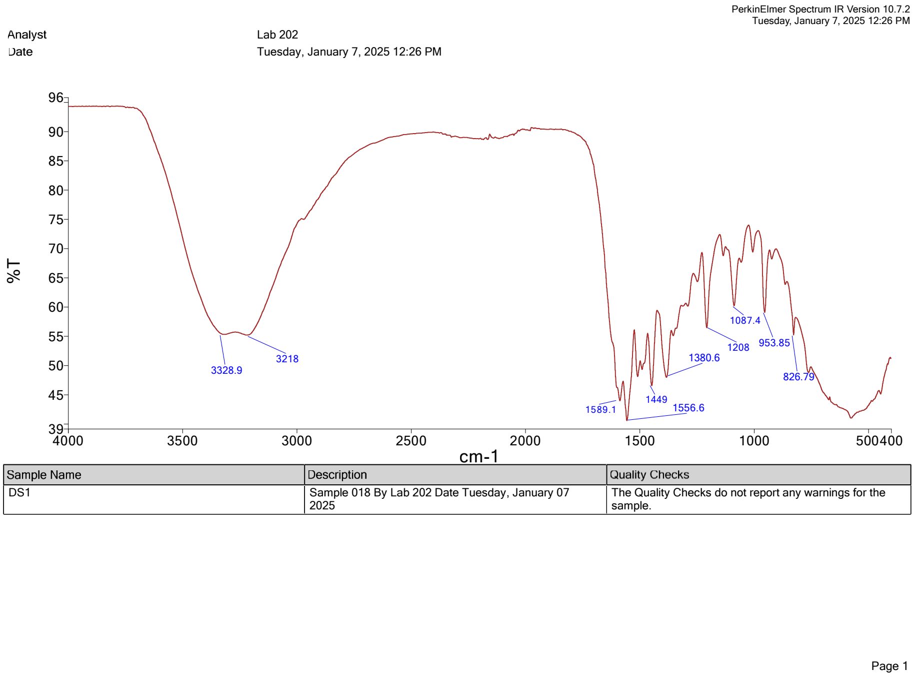

Fig.S27 – ATF-FTIR spectra of $[Choline]_2[MTX]$

PerkinElmer Spectrum IR Version 10.7.2
Tuesday, January 7, 2025 12:25 PM

Analyst Lab 202
Date Tuesday, January 7, 2025 12:25 PM

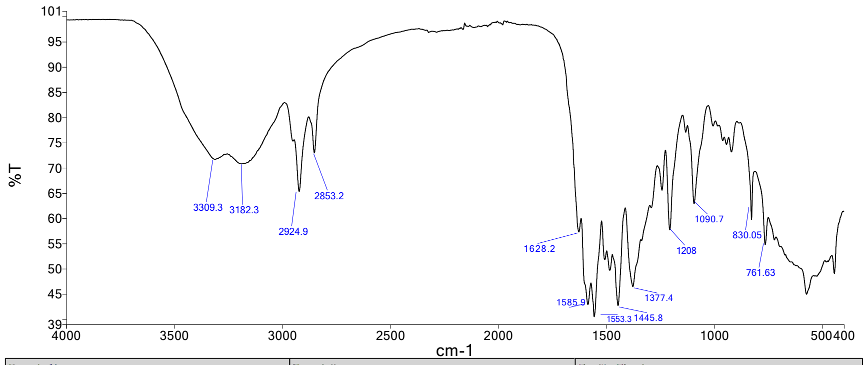

DS28	Sample 011 By Lab 202 Date Tuesday, January 07 2025	The Quality Checks do not report any warnings for the sample.

Page 1

Fig.S28 ATF-FTIR spectra of $[N_{12,1,1,2OH}]_2[MTX]$

PerkinElmer Spectrum IR Version 10.7.2
Tuesday, January 7, 2025 12:25 PM

Analyst Lab 202
Date Tuesday, January 7, 2025 12:25 PM

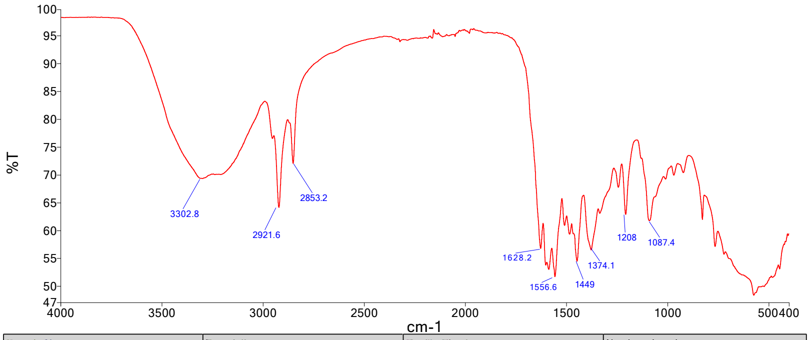

			Number of peaks
DS27	Sample 008 By Lab 202 Date Tuesday, January 07 2025	The Quality Checks do not report any warnings for the sample.	18

Page 1

Fig.S29 - ATF-FTIR spectra of $[N_{10,1,1,2OH}]_2[MTX]$

PerkinElmer Spectrum IR Version 10.7.2
Tuesday, January 7, 2025 12:26 PM

Analyst Lab 202
Date Tuesday, January 7, 2025 12:26 PM

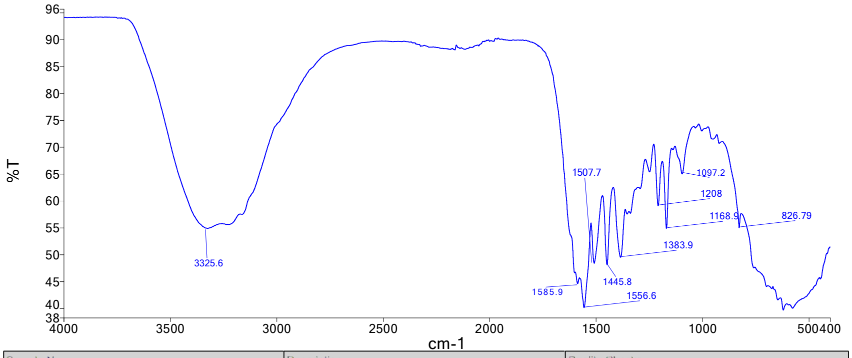

DS10	Sample 016 By Lab 202 Date Tuesday, January 07 2025	The Quality Checks do not report any warnings for the sample.

Page 1

Fig.S30 - ATF-FTIR spectra of $[C_2mim]_2[MTX]$

PerkinElmer Spectrum IR Version 10.7.2
Tuesday, January 7, 2025 12:48 PM

Analyst Lab 202
Date Tuesday, January 7, 2025 12:48 PM

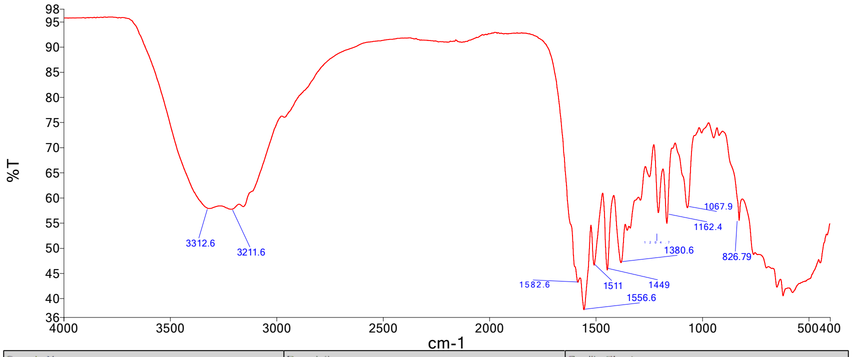

DS3	Sample 008 By Lab 202 Date Tuesday, January 07 2025	The Quality Checks do not report any warnings for the sample.

Page 1

Fig.S31 - ATF-FTIR spectra of $[C_2OHmim]_2[MTX]$

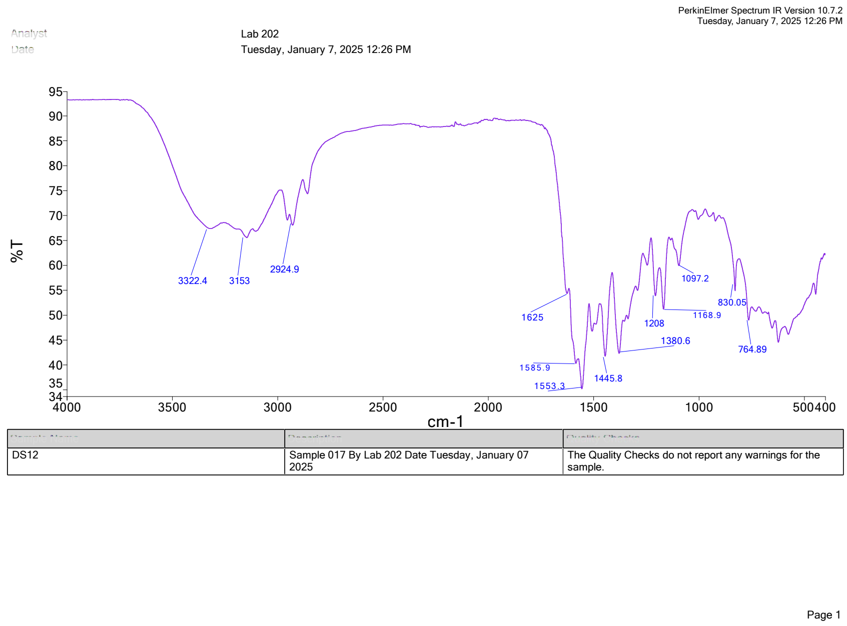

Fig.S32 - ATF-FTIR spectra of $[C_6mim]_2[MTX]$

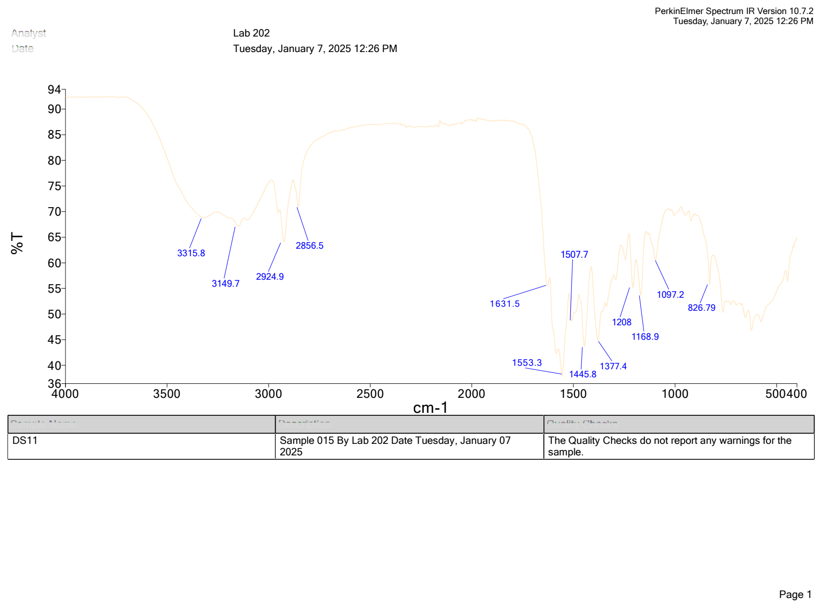

Fig.S33 - ATF-FTIR spectra of $[C_8mim]_2[MTX]$

PerkinElmer Spectrum IR Version 10.7.2
Tuesday, January 7, 2025 12:26 PM

Analyst	Lab 202
Date	Tuesday, January 7, 2025 12:26 PM

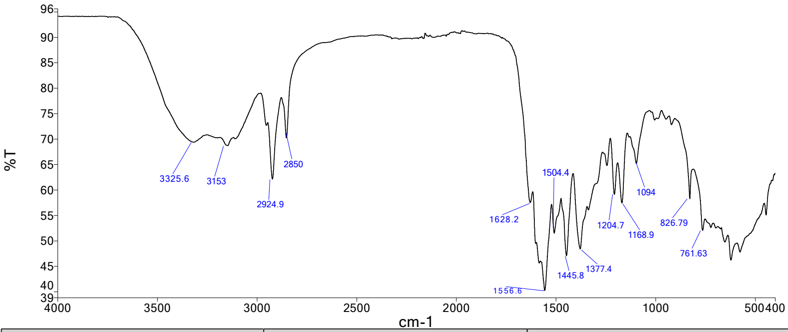

DS4	Sample 014 By Lab 202 Date Tuesday, January 07 2025	The Quality Checks do not report any warnings for the sample.

Page 1

Fig.S34 - ATF-FTIR spectra of [C$_{10}$mim]$_2$[MTX]

PerkinElmer Spectrum IR Version 10.7.2
Tuesday, January 7, 2025 12:26 PM

Analyst	Lab 202
Date	Tuesday, January 7, 2025 12:26 PM

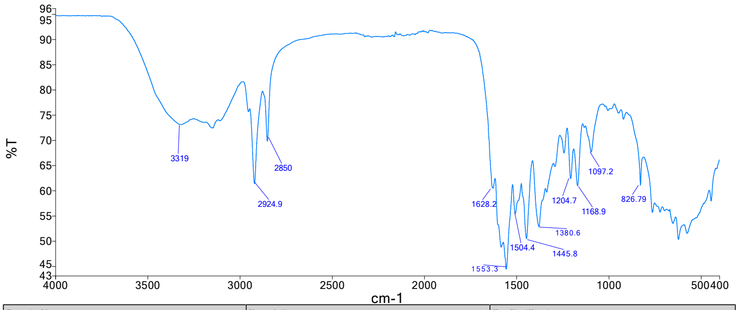

DS2	Sample 013 By Lab 202 Date Tuesday, January 07 2025	The Quality Checks do not report any warnings for the sample.

Page 1

Fig.S35 - ATF-FTIR spectra of [C$_{12}$mim]$_2$[MTX]

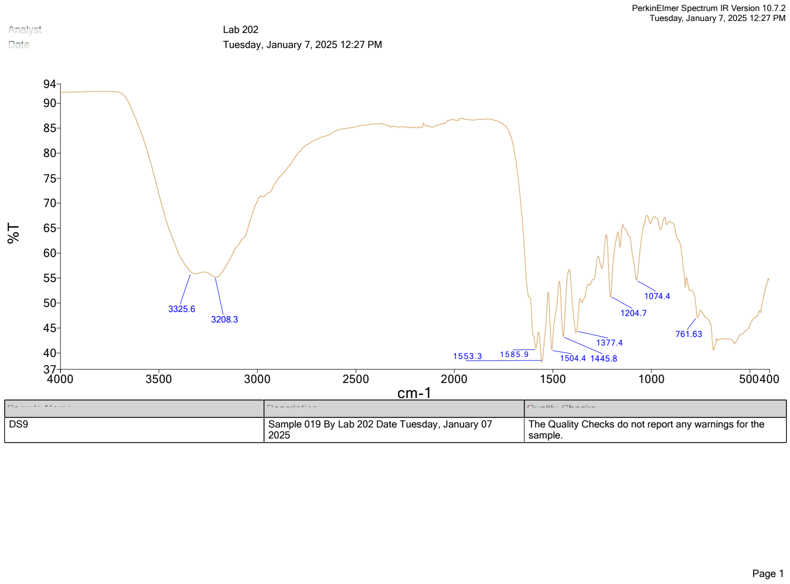

Fig.S36 - ATF-FTIR spectra of [C_2OH 3-picoline]$_2$[MTX]

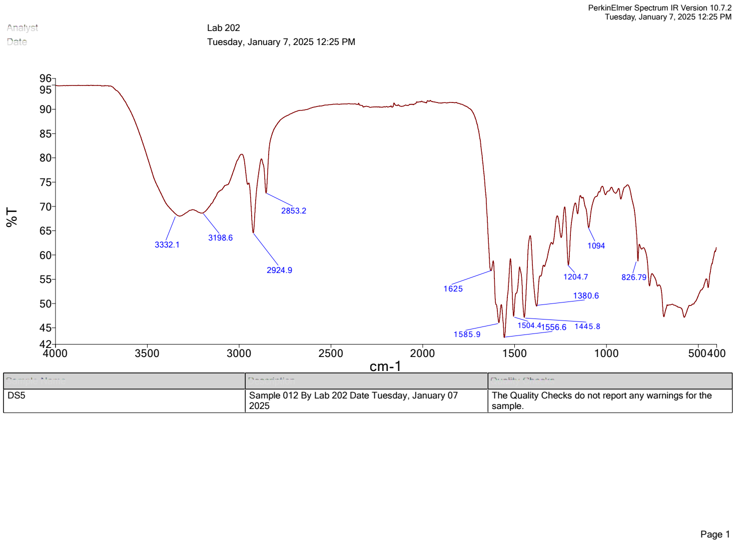

Fig.S37 - ATF-FTIR spectra of [C_{10} 3-picoline]$_2$[MTX]

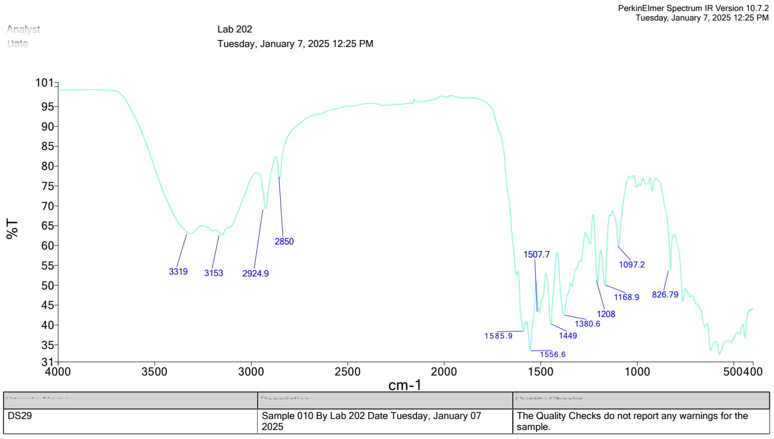

Fig.S38 - ATF-FTIR spectra of $[MIMC_{12}MIM]_2[MTX]$

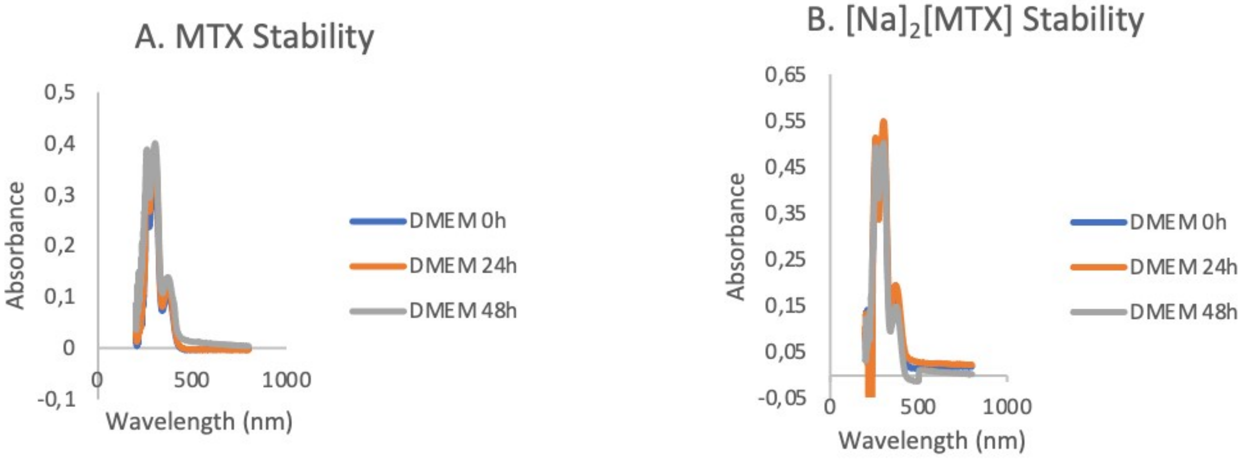

Fig.S39 - UV/Vis spectrum of A. MTX and B. $[Na]_2[MTX]$ in DMEM after 0, 24 and 48h at 37 ^{0}C

Biological Studies

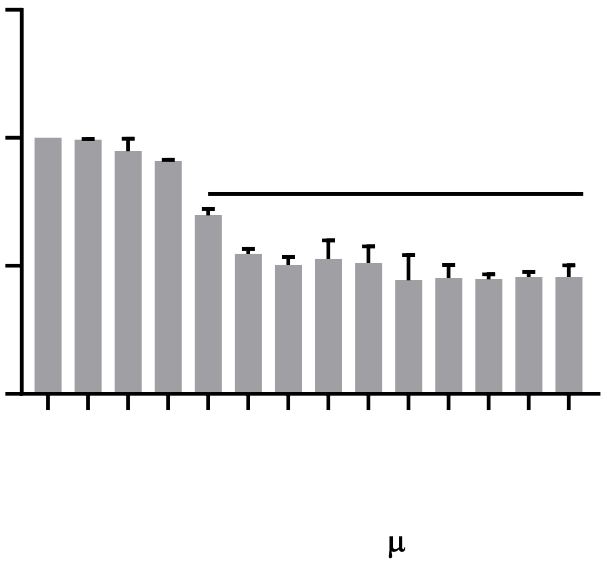

Fig.S40 - Cell viability of A549 cell line after exposure to different concentrations of the $[Na]_2[MTX]$ for 48 hours. 0.1% (v/v) DMSO was used as the vehicle control. (**** $p < 0.0001$).

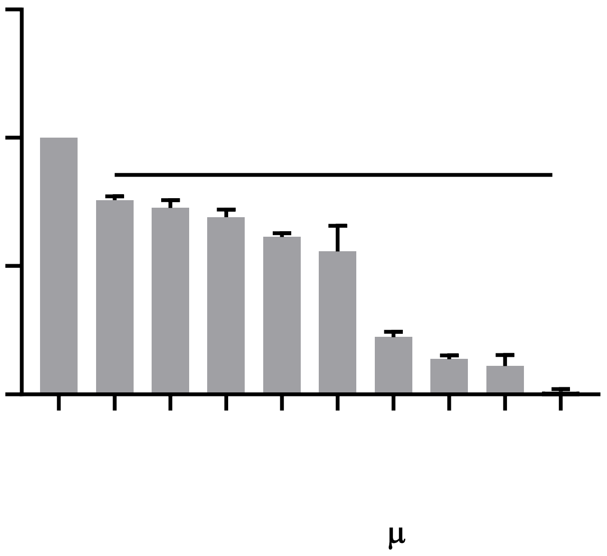

Fig.S41 - Cell viability of A549 cell line after exposure to different concentrations of the $[N_{12,1,1,2OH}]_2[MTX]$ for 48 hours. 0.1% (v/v) DMSO was used as the vehicle control. (**** $p < 0.0001$).

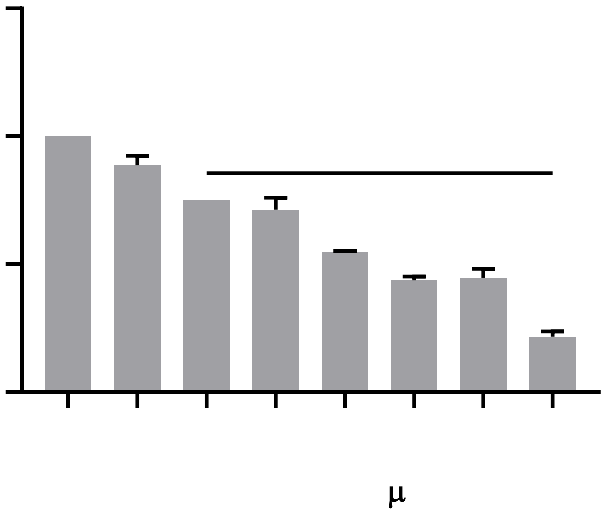

Fig.S42 - Cell viability of A549 cell line after exposure to different concentrations of the $[N_{10,1,1,2OH}]_2$[MTX] for 48 hours. 0.1% (v/v) DMSO was used as the vehicle control. (*** p < 0.001; **** p < 0.0001).

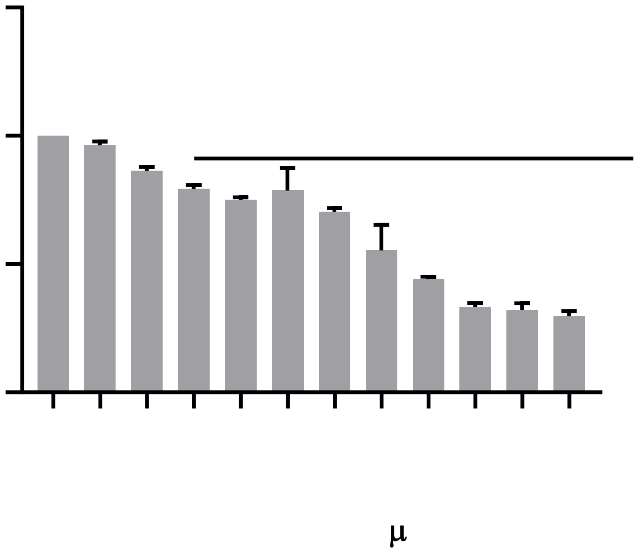

Fig.S43 - Cell viability of A549 cell line after exposure to different concentrations of the $[MIMC_{12}MIM]_2$[MTX] for 48 hours. 0.1% (v/v) DMSO was used as the vehicle control. (** p < 0.01; **** p < 0.0001).

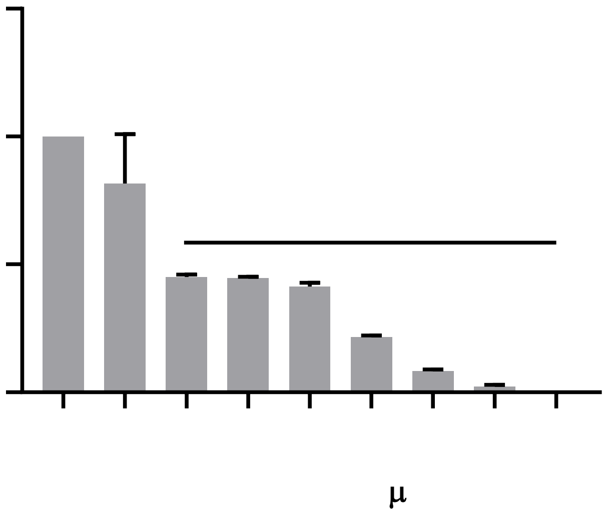

Fig.S44 - Cell viability of A549 cell line after exposure to different concentrations of the [C_{12}mim][Br] for 48 hours. 0.1% (v/v) DMSO was used as the vehicle control. (* p < 0.05; **** p < 0.0001).

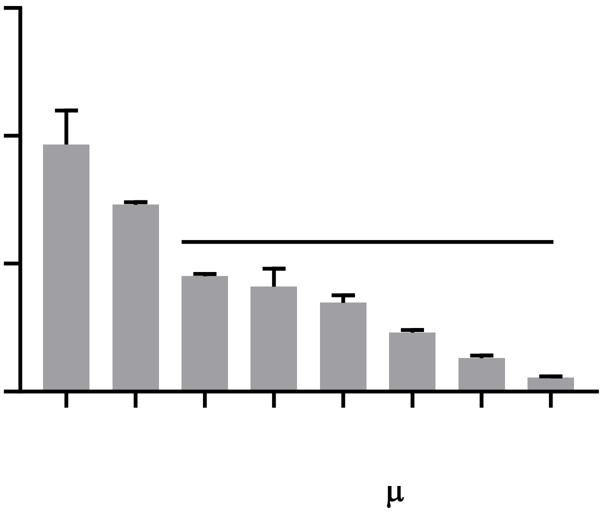

Fig.S45 - Cell viability of A549 cell line after exposure to different concentrations of the [C_{10}mim][Br] for 48 hours. 0.1% (v/v) DMSO was used as the vehicle control. (*** p < 0.001; **** p < 0.0001).

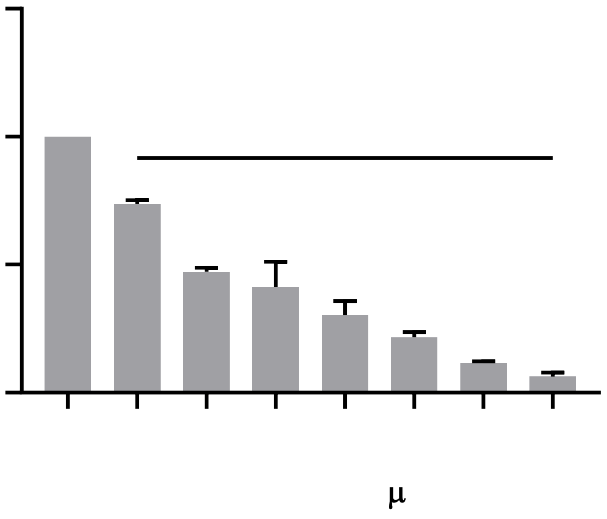

Fig.S46 - Cell viability of A549 cell line after exposure to different concentrations of the [C_{10} 3-picoline][Br] for 48 hours. 0.1% (v/v) DMSO was used as the vehicle control. (**** $p < 0.0001$).

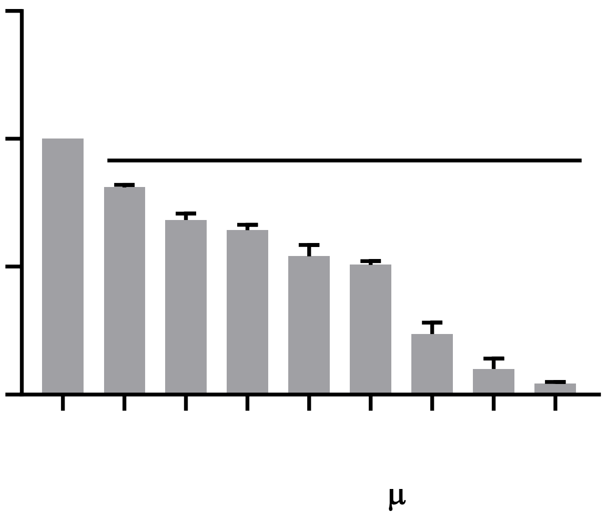

Fig.S47 - Cell viability of H1975 cell line after exposure to different concentrations of the [C_{12}mim]$_2$[MTX] for 48 hours. 0.1% (v/v) DMSO was used as the vehicle control. (**** $p < 0.0001$).

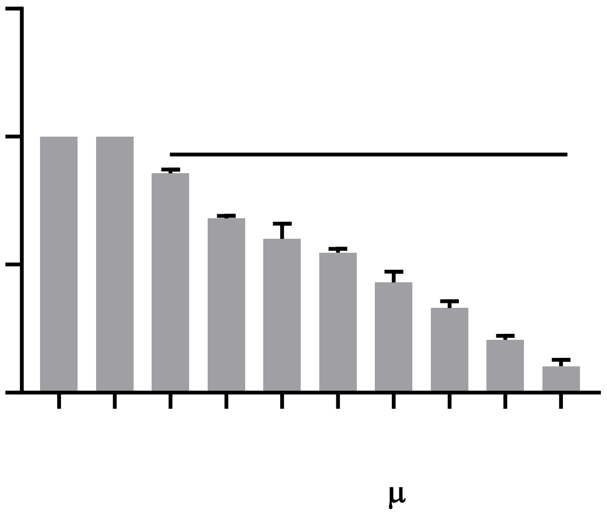

Fig.S48 - Cell viability of H1975 cell line after exposure to different concentrations of the $[C_{10}mim]_2[MTX]$ for 48 hours. 0.1% (v/v) DMSO was used as the vehicle control. (**** $p < 0.0001$).

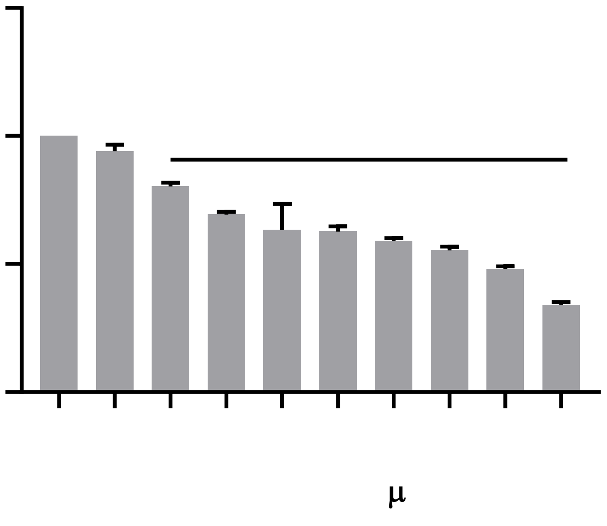

Fig.S49 - Cell viability of H1975 cell line after exposure to different concentrations of the $[C_{10}$ 3-picoline$]_2[MTX]$ for 48 hours. 0.1% (v/v) DMSO was used as the vehicle control. (**** $p < 0.0001$).

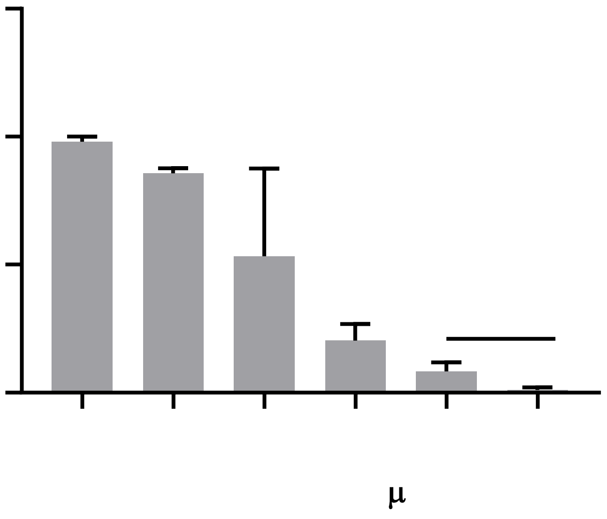

Fig.S50 - Cell viability of H1975 cell line after exposure to different concentrations of the [C_{12}mim][Br] for 48 hours. 0.1% (v/v) DMSO was used as the vehicle control. (* $p < 0.05$; ** $p < 0.01$; **** $p < 0.0001$).

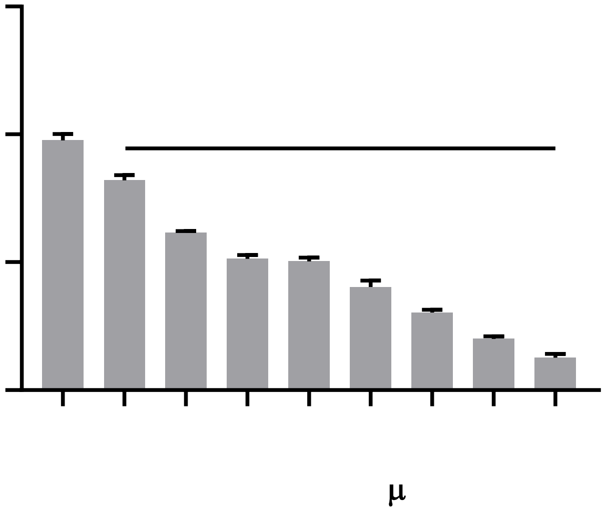

Fig.S51 - Cell viability of H1975 cell line after exposure to different concentrations of the [C_{10}mim][Br] for 48 hours. 0.1% (v/v) DMSO was used as the vehicle control. (**** $p < 0.0001$).

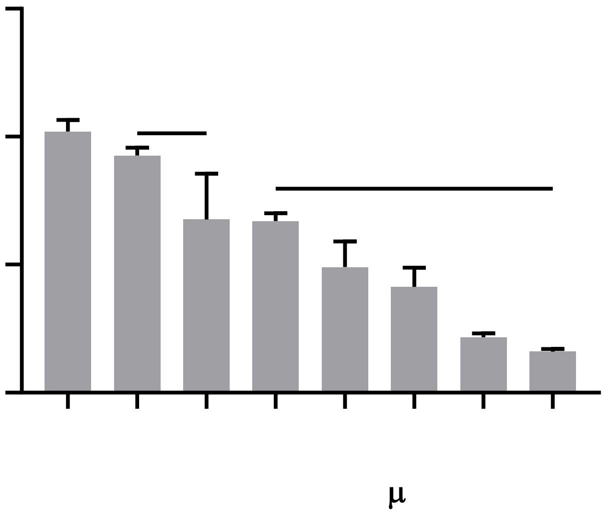

Fig.S52 - Cell viability of H1975 cell line after exposure to different concentrations of the [C_{10} 3-picoline][Br] for 48 hours. 0.1% (v/v) DMSO was used as the vehicle control. (*** p < 0.001; **** p < 0.0001).

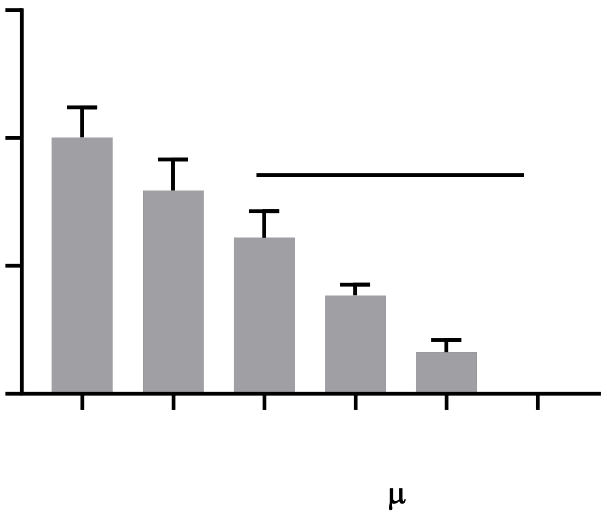

Fig.S52 - Cell viability of Fibroblasts after exposure to different concentrations of the [C_{12}mim]$_2$[MTX] for 48 hours. 0.1% (v/v) DMSO was used as the vehicle control. (* p < 0.05; **** p < 0.0001).

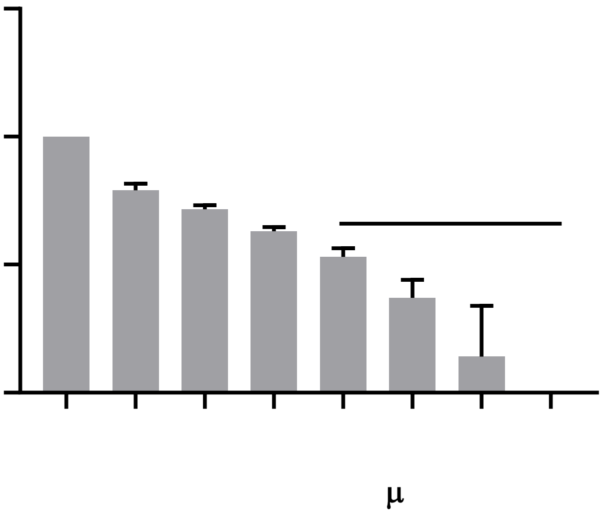

Fig.S53 - Cell viability of Fibroblasts after exposure to different concentrations of the $[C_{10}mim]_2[MTX]$ for 48 hours. 0.1% (v/v) DMSO was used as the vehicle control. (* $p < 0.05$; ** $p < 0.01$; *** $p < 0.001$; **** $p < 0.0001$).

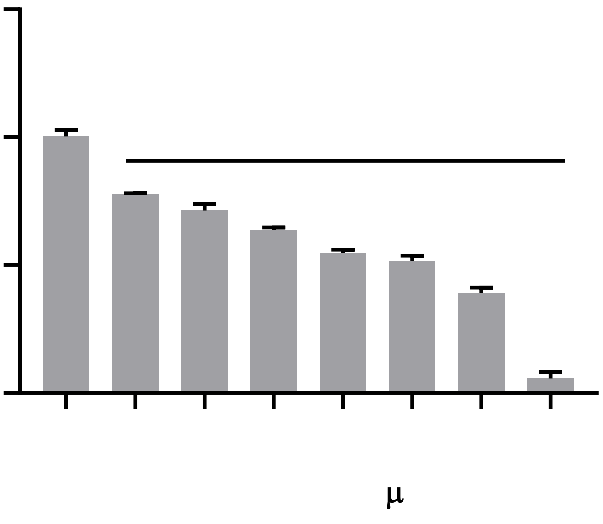

Fig.S53 - Cell viability of Fibroblasts after exposure to different concentrations of the $[C_{10}$ 3-picoline$]_2[MTX]$ for 48 hours. 0.1% (v/v) DMSO was used as the vehicle control. (**** $p < 0.0001$).

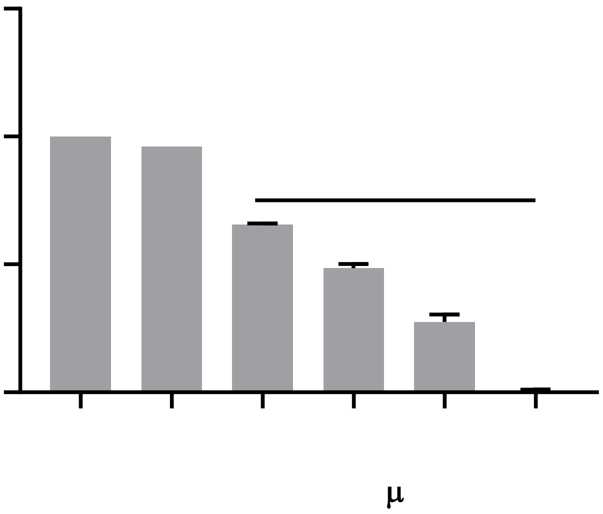

Fig.S54 - Cell viability of Fibroblasts after exposure to different concentrations of the [C_{12}mim][Br] for 48 hours. 0.1% (v/v) DMSO was used as the vehicle control. (** $p < 0.01$; **** $p < 0.0001$).

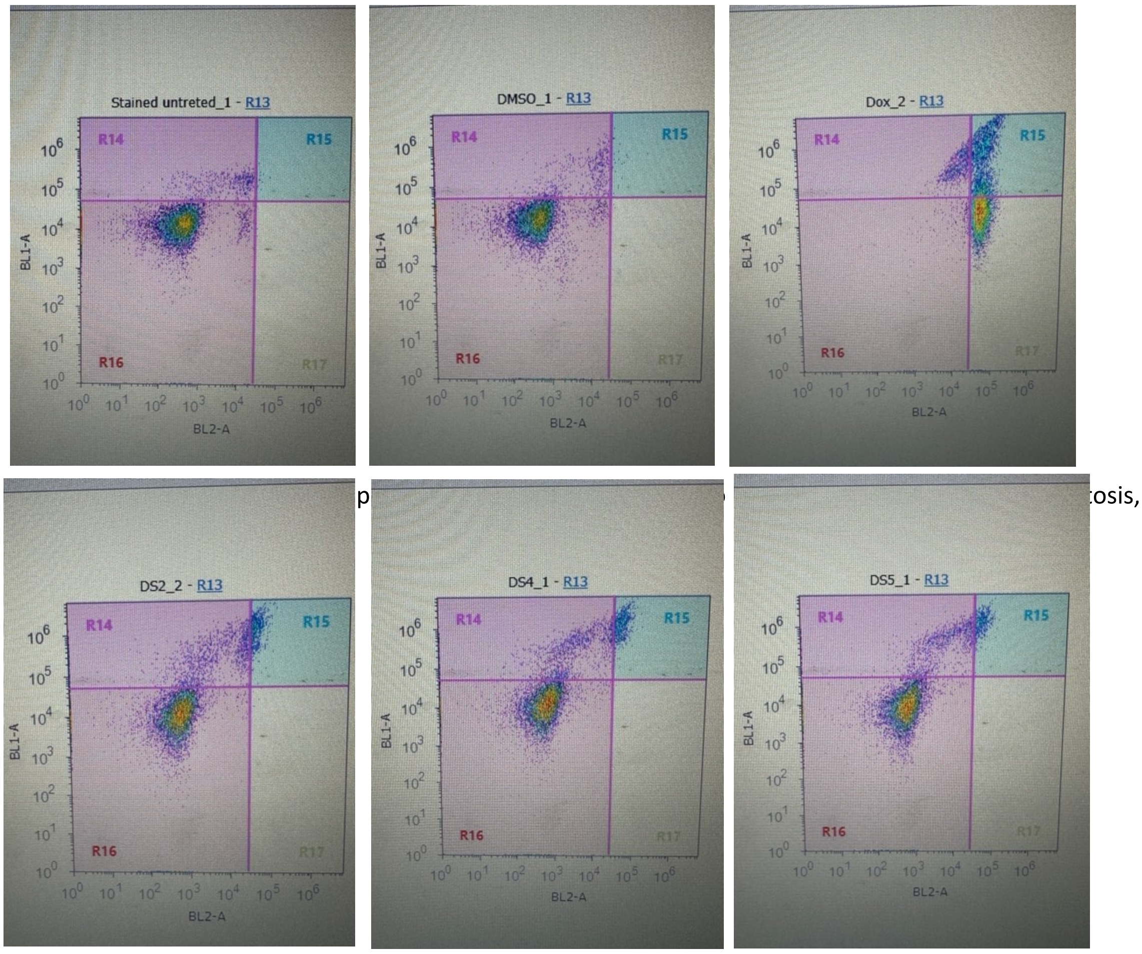

p [illegible] tosis,

and R17 cells in necrosis. DS2 correspond to [C_{12}mim] $_2$[MTX], DS4 correspond to [C_{10}mim] $_2$[MTX] and DS5 correspond to [C_{10} 3-picoline]$_2$ [MTX].
